# Clinical manifestations of Oropouche virus infection: A systematic review and meta-analysis

**DOI:** 10.3892/mi.2025.266

**Published:** 2025-08-27

**Authors:** Mohan Giri, Anju Puri, Shiv Kumar Yadav, Yi Chen

**Affiliations:** 1 Department of Respiratory and Critical Care Medicine, The First Affiliated Hospital of Chongqing Medical University, Chongqing 400016, P.R. China; 2 Department of Nursing, The First Affiliated Hospital of Chongqing Medical University, Chongqing 400016, P.R. China; 3 Department of Pediatric Cardiology, Children's Hospital of Chongqing Medical University, Chongqing 400014, P.R. China

**Keywords:** Oropouche virus, clinical manifestations, arbovirus, prevalence, symptoms

## Abstract

Oropouche virus (OROV) is emerging as a growing public health concern, with increasing numbers of case, an expanding global spread and the potential for severe clinical outcomes. However, despite the increasing incidence, the clinical features of OROV infections have not yet been thoroughly examined. The present systematic review and meta-analysis aimed to investigate the prevalence of clinical manifestations in OROV infections. For this purpose, a comprehensive search across PubMed, Web of Science and Embase was conducted up to April 9, 2025, to identify relevant studies. A random effects model was employed to calculate the pooled prevalence and 95% confidence intervals were calucalted. Heterogeneity was assessed using the I^2^ statistic. Additionally, sensitivity analyses and publication bias assessments were conducted to ensure the robustness of our findings. The present study included 28 articles and assessed 4,196 patients with OROV infection from 6 countries across the globe. The pooled prevalence of clinical manifestations of OROV included fever (97%), headache (86.5%), myalgia (72.3%), malaise or fatigue (56.4%), arthralgia (50.3%), chills (49.6%), loss of appetite (44.3%), eye pain (43.2%), back pain (31.7%), pallor (31.7%), dizziness (30.2%), photophobia (30.9%), nausea/vomiting (28.9%), sore throat (26.1%), odynophagia (22.9%), diarrhea (18.4%), skin rash (18.2%), conjunctival injection (15.4%), abdominal pain (16.3%), petechiae (2.3%), cough (12.9%), and chest pain (0.7%). High heterogeneity was detected among the included studies, which may be attributed to differences in geographic locations and diagnostic methodologies. Sensitivity analyses further supported the robustness of our findings. On the whole, the present systematic review provides a comprehensive analysis of the clinical manifestations of OROV infection, highlighting key symptoms that may aid in differential diagnosis in arbovirus-endemic regions. The findings may provide critical insight for clinicians and public health professionals and lay the groundwork for future research on the pathogenesis and epidemiology of OROV.

## Introduction

The Oropouche virus (OROV), an arbovirus of growing public health concern, is mainly spread through the bite of *Culicoides paraensis* midges, with mosquitoes playing a secondary role in transmission (1). OROV is a member of the *Orthobunyavirus* genus within the *Peribunyaviridae* family and possesses a segmented, single-stranded negative-sense RNA genome approximately 12.5 kb in length (1). The virus has been responsible for recurrent outbreaks of acute febrile illness across tropical and subtropical regions of Latin America, particularly in Brazil, Peru and the Amazon basin (2-4). First identified in Trinidad in 1955, the OROV has since been responsible for numerous human infections. Its transmission involves a zoonotic cycle, with sloths and primates serving as reservoirs, while humans are typically incidental hosts (1). Recent epidemiological surveillance indicates its expansion into previously unaffected geographic regions, raising significant concerns about its potential for broader dissemination (5).

The symptoms of Oropouche fever often mirror those of other arboviral infections, including dengue fever, chikungunya virus and Zika virus, posing challenges for accurate clinical differentiation in regions where these viruses co-circulate (1,6). The clinical presentation of OROV often features rapid fever development together with cephalgia, muscular pain, arthralgic symptoms and ocular photophobia. Neurological manifestations, such as cerebral inflammation or meningeal syndrome may occur in severe presentations (1,4). Although the OROV causes a substantial disease burden, it is often underdiagnosed and underreported, primarily due to restricted diagnostic capabilities and the similarity of its symptoms with other febrile conditions (7).

To date, no comprehensive synthesis of the clinical manifestations of OROV infection has been conducted across diverse outbreak settings and populations. The clinical spectrum of OROV infection remains incompletely characterized, necessitating a comprehensive synthesis of existing evidence. A systematic review and meta-analysis represent a critical approach to consolidating epidemiological and clinical data, which will facilitate the identification of predominant symptom patterns and their prevalence. Such an analysis is vital for refining diagnostic criteria and optimizing patient management in endemic regions.

The present aimed to systematically evaluate the clinical manifestations of OROV infection by conducting a meta-analysis of published literature. This research addresses existing knowledge gaps and strengthens evidence-based clinical and public health interventions by elucidating the symptomatic profile and disease burden. The findings provided herein may provide a foundation for improved case detection, surveillance, and therapeutic decision-making in areas affected by this emerging arbovirus.

## Data and methods

The present systematic review and meta-analysis strictly adhered to the Preferred Reporting Items for Systematic Reviews and Meta-Analyses (PRISMA) guidelines (8).

### Search strategy

For the search, two authors (MG and AP) independently performed a systematic literature search of electronic databases, including PubMed, Web of Science and Embase, to identify studies reporting the clinical manifestations of OROV infection from inception until April 9, 2025, without language restrictions. Both peer-reviewed and available preprint publications were considered for inclusion. A systematic literature search was independently conducted by two authors (MG and AP), using a combination of terms related to the Oropouche virus, including ‘Oropouche virus’, ‘Oropouche fever’, ‘Oropouche orthobunyavirus’, ‘Oropouche virus infection’, ‘Oropouche virus disease’, ‘Orthobunyavirus Oropouche’ and ‘OROV’. The detailed search strategy for each database is presented in Table SI. The references of relevant articles were manually reviewed to identify additional studies.

### Eligibility criteria

The inclusion criteria for studies in the present systematic review and meta-analysis were as follows: i) Studies involving patients with laboratory-confirmed OROV infection, with confirmation established through RT-PCR or serological assays; ii) studies that provided detailed descriptions of the clinical features associated with OROV infection. Eligible study designs included cohort, case-control, cross-sectional studies and case series. The exclusion criteria were the following: i) Studies with <5 participants; ii) research conducted on animals; iii) non-original publications such as reviews, meta-analyses, commentaries, errata and other editorial content; and iv) studies lacking comprehensive clinical data.

### Study selection and data extraction

Two authors (MG and AP) independently conducted study selection and data extraction. Titles and abstracts of all retrieved records were screened to determine eligibility according to predefined inclusion criteria. Any discrepancies were resolved through discussion, with input from a third reviewer when necessary. Data were extracted using pre-designed Excel forms for all studies meeting the eligibility criteria to ensure standardized and systematic collection. Extracted variables included study setting (country), study design, population characteristics, study period, sample size, diagnostic methods, and reported clinical manifestations.

### Quality assessment

The methodological quality of the included observational studies was evaluated using a simplified and modified version of the Newcastle-Ottawa Scale (NOS) (9). This version assessed four key domains: The representativeness of the study population, adequacy of the sample size, clarity and validity of the diagnostic criteria used to confirm OROV infection, and the reliability of outcome ascertainment. Each study was assigned a total score ranging from 0 to 6, with studies categorized as low (score 0-2), moderate (score 3-4), or high quality (score 5-6). For case series and case reports, the methodological quality was evaluated according to the framework proposed in the study by Murad *et al* (10). The results of the quality appraisal for all included studies are presented in Tables SII and SIII.

### Statistical analysis

All statistical analyses were performed using R software, version 4.4.1, with the ‘meta’ and ‘metafor’ packages for Windows. A Freeman-Tukey double arcsine transformation with a random-effects model was applied to compute the weighted proportion of clinical manifestations and corresponding 95% confidence intervals (CIs). Given the observed variability between the included studies, DerSimonian and Laird inverse-variance-weighted random-effects models were used. The I^2^ statistic and Cochran's Q test were used to assessed the heterogeneity of the included studies. The I^2^ values were categorized as low (<25%), moderate (25-75%) and high (>75%) to indicate the level of heterogeneity. Publication bias was evaluated visually via funnel plots and quantitatively using Begg's regression test. A sensitivity analysis was also performed through a leave-one-out meta-analysis to assess each study's effect on the overall pooled estimates. All statistical tests were two-sided, with P-values of less than 0.05 considered statistically significant.

## Results

### Search results and characteristics of the included studies

**Figure 1:**
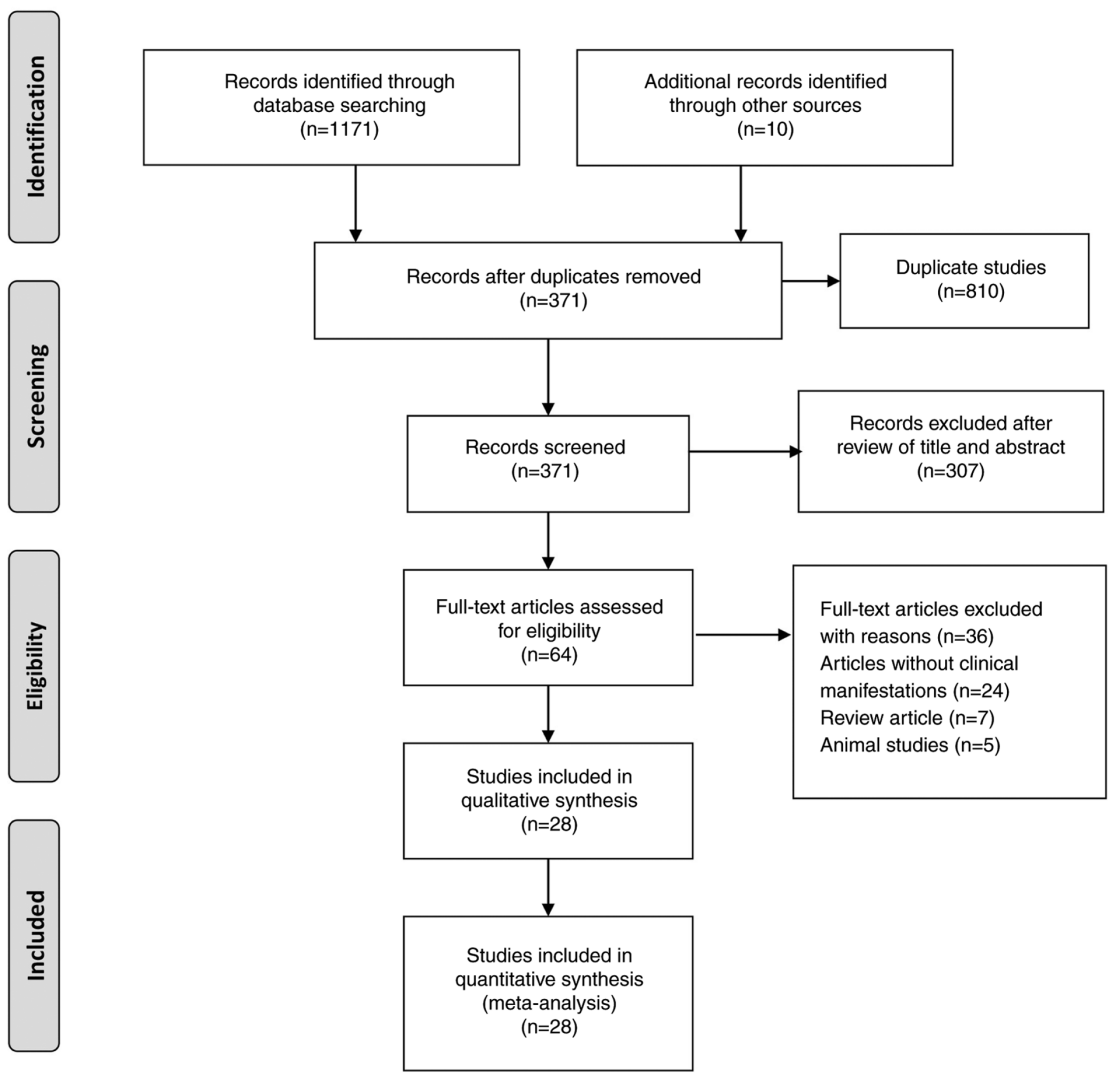
PRISMA flowchart of literature search for the clinical characteristics of Oropouche virus infection.

A total of 1,171 records were retrieved from the databases searched, with an additional 10 records identified from other sources. After removing duplicates, 371 records remained for eligibility assessment. After excluding studies that did not meet the predefined inclusion criteria, 28 articles were selected for inclusion in the present systematic review, encompassing a total sample size of 4,196 participants. The study selection process is illustrated in Fig. 1. Of these, 26 were observational studies, and two studies were case series. An overview of the 28 included articles revealed that 14 studies were conducted in Brazil (2,11-23), eight in Peru (24-31), two in France (32,33), two in Cuba (34,35), one in Colombia (36), and one study in the USA (37). The sample sizes of the included studies varied considerably, ranging from as few as 5 participants to as many as 2,272, highlighting the diverse scale and scope of the OROV research. The studies were conducted between 1976 and 2025. The detailed characteristics of the 28 included studies are presented in Table I.

### Methodological quality of the included studies

The overall methodological quality of the majority of the studies, as assessed using the modified Newcastle-Ottawa Scale, was high, with a score of ≥5. A total of three studies were classified as moderate quality (with a score of 4). A summayr of the Newcastle-Ottawa Scale scores for each study, along with a brief overview of each item, is presented in Table SII. Furthermore, the methodological quality assessment of the case series was also of high quality (Table SIII).

*Meta-analyses of prevalence: General or systemic manifestations*

### Fever

**Figure 2:**
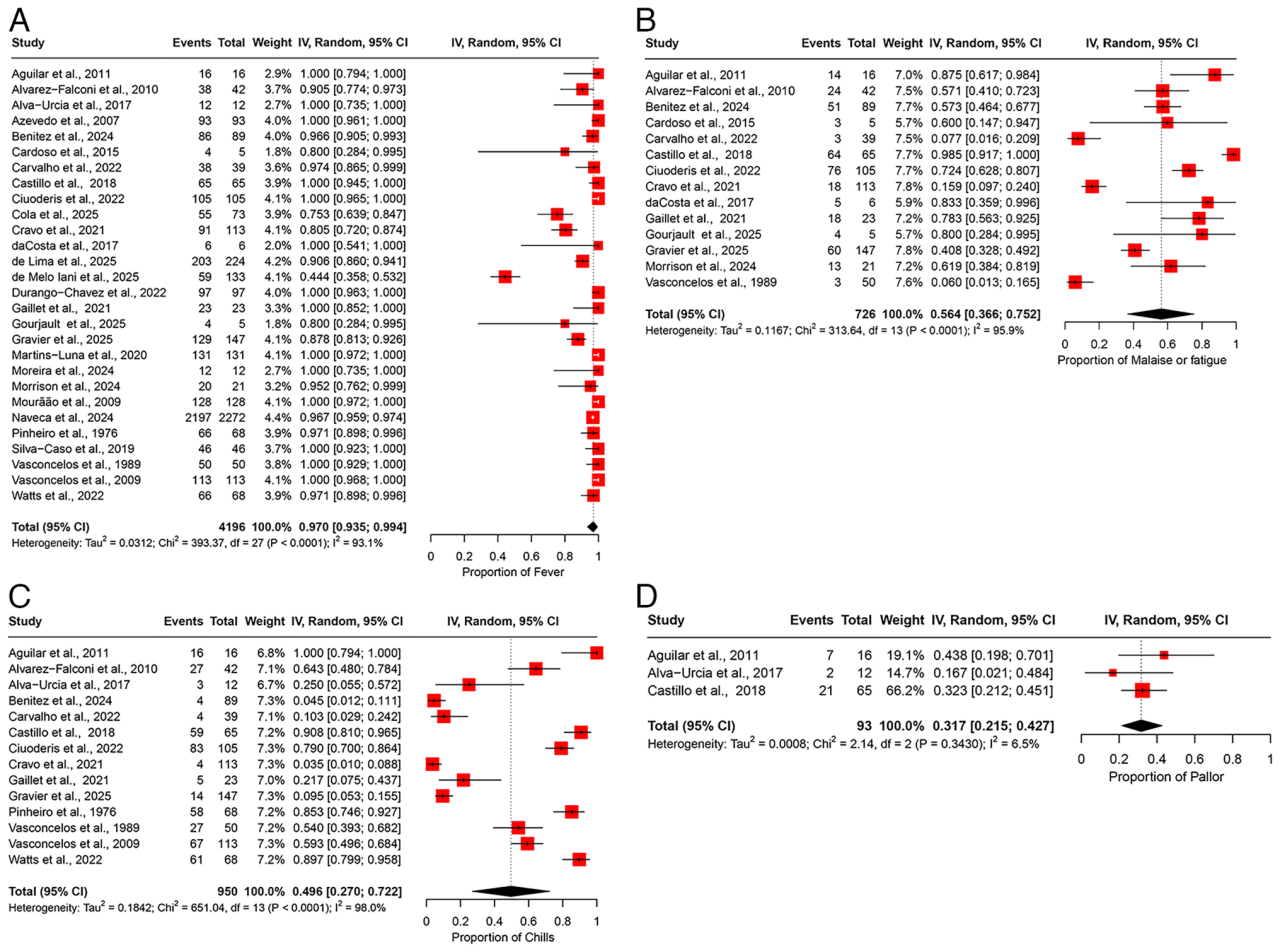
Forest plot of the prevalence of general or systemic manifestations among patients infected with OROV. (A) Pooled prevalence of fever among OROV-infected patients. (B) The pooled prevalence of malaise or fatigue among OROV-infected patients . (C) The pooled prevalence of chills among OROV-infected patients. (D) Prevalence of pallor among OROV-infected patients. The studies included were as follows: Aguilar *et al* (24), Alvarez-Falconi *et al* (25), Alva-Urcia *et al* (26), Azevedo *et al* (11), Benitez *et al* (34), Cardoso *et al* (16), Carvalho *et al* (17), Castillo *et al* (27), Ciuoderis *et al* (36), Cola *et al* (18), Cravo *et al* (19), da Costa *et al* (20), de Lima *et al* (21), de Melo Iani *et al* (22), Durango-Chavez *et al* (28), Gaillet *et al* (32), Gourjault *et al* (33), Gravier *et al* (35), Martins-Luna *et al* (29), Moreira *et al* (2), Morrison *et al* (37), Mourão *et al* (23), Naveca *et al* (12), Pinheiro *et al* (13), Silva-Caso *et al* (30), Vasconcelos *et al* (14), Vasconcelos *et al* (15), Watts *et al* (31) OROV, Oropouche virus; 95% CI, 95% confidence interval.

The pooled prevalence of fever among patients infected with OROV was 97.0% (95% CI, 93.5-99.4%) across 28 studies, encompassing a total of 4,196 participants (Fig. 2A). The heterogeneity across studies was substantial, with an I^2^ value of 93.1%, indicating significant variability in the reported prevalence rates between studies (Table II). A sensitivity analysis was performed by sequentially omitting one study at a time. The pooled prevalence of fever remained consistent, indicating that no individual study affected the overall result, demonstrating the robustness of the findings (Fig. S1A).

*Malaise or fatigue*. The pooled prevalence of malaise or fatigue across 14 studies involving 726 participants was 56.4% (95% CI, 36.6-75.2%) (Fig. 2B). An I^2^ value of 95.9% indicated substantial heterogeneity in the prevalence estimates reported across the studies (Table II). A sensitivity analysis was conducted by sequentially omitting one study at a time from the meta-analysis. The pooled prevalence estimates remained relatively stable, ranging from 53.7% (95% CI, 33.4-3.5%) to 61.3% (95% CI, 41.7-79.3%) (Fig. S1B). These results suggest that no single study significantly affected the overall pooled prevalence estimate, indicating the robustness of our findings.

*Chills*. The pooled prevalence of chills was 49.6% (95% CI, 27.0-72.2%) among patients with OROV infection, based on data from 950 patients across 14 studies (Fig. 2C). The heterogeneity in the prevalence of chills was high, with an I^2^ value of 98%, suggesting considerable differences in the rates of chills across studies (Table II). The results of the sensitivity analysis, in which one study was excluded at a time, demonstrated that the overall prevalence estimate of chills remained stable and was not significantly impacted by leaving one study at a time (Fig. S1C).

*Pallor*. Only three studies provided data on the prevalence of pallor, yielding a pooled prevalence of 31.7% (95% CI, 21.5-42.7%) with a total of 93 participants (Fig. 2D). The studies exhibited low heterogeneity, with an I^2^ value of 6.5%, suggesting consistency in the results (Table II). A sensitivity analysis, performed by sequentially removing one study at a time, demonstrated that the pooled prevalence estimate remained stable and was not significantly influenced by the exclusion of any individual study (Fig. S1D).

### Meta-analyses of prevalence: Neurological manifestations. Headache

**Figure 3:**
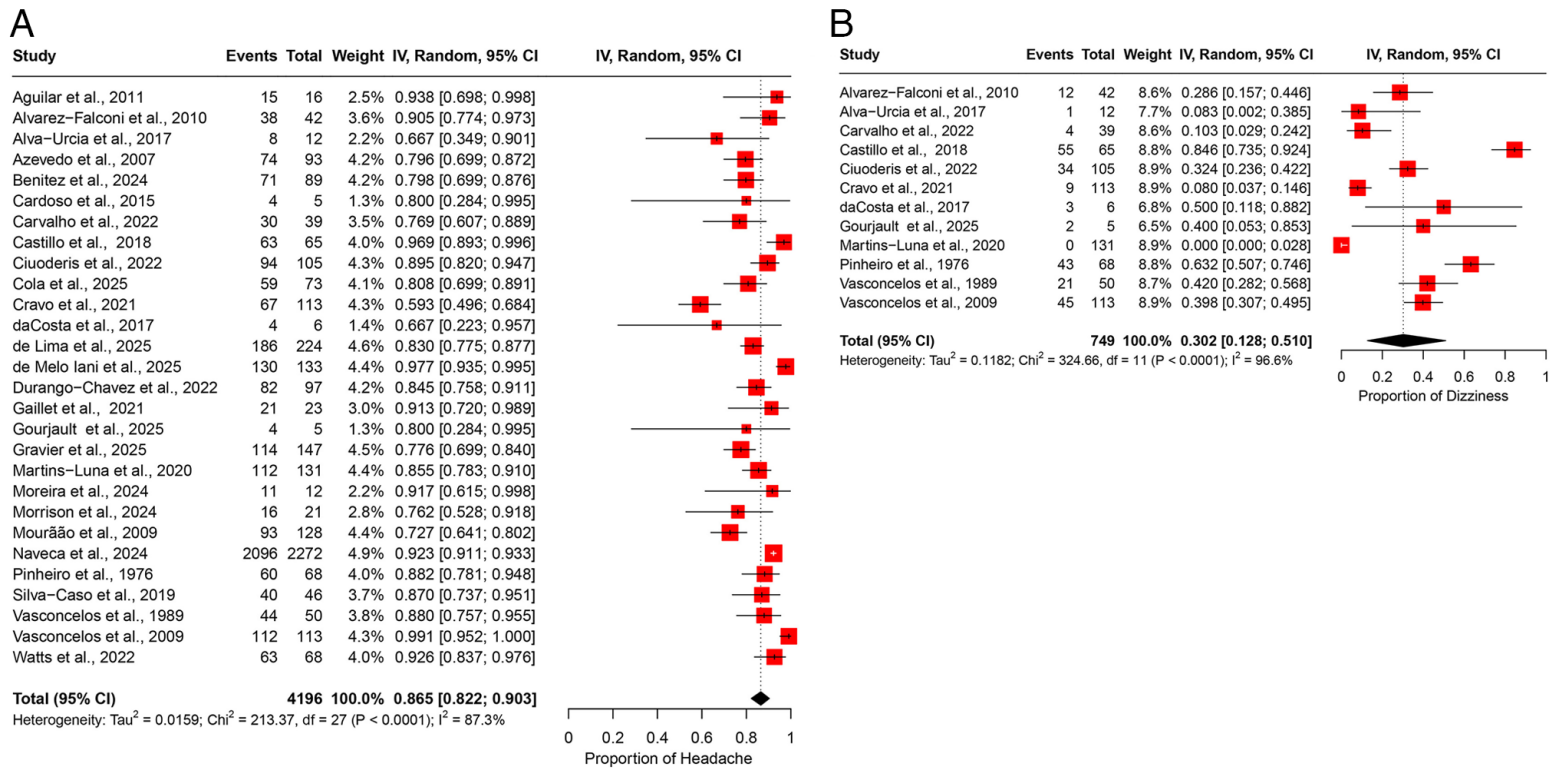
Forest plot of the prevalence of neurological manifestations among patients infected with OROV. (A) The pooled prevalence of headache among OROV-infected patients. (B) The pooled prevalence of dizziness among OROV-infected patients. The studies included were as follows: Aguilar *et al* (24), Alvarez-Falconi *et al* (25), Alva-Urcia *et al* (26), Azevedo *et al* (11), Benitez *et al* (34), Cardoso *et al* (16), Carvalho *et al* (17), Castillo *et al* (27), Ciuoderis *et al* (36), Cola *et al* (18), Cravo *et al* (19), da Costa *et al* (20), de Lima *et al* (21), de Melo Iani *et al* (22), Durango-Chavez *et al* (28), Gaillet *et al* (32), Gourjault *et al* (33), Gravier *et al* (35), Martins-Luna *et al* (29), Moreira *et al* (2), Morrison *et al* (37), Mourão *et al* (23), Naveca *et al* (12), Pinheiro *et al* (13), Silva-Caso *et al* (30), Vasconcelos *et al* (14), Vasconcelos *et al* (15), Watts *et al* (31). OROV, Oropouche virus; 95% CI, 95% confidence interval.

A meta-analysis of 28 studies, including 4,196 patients with OROV infection, estimated the polled prevalence of headaches at 86.5% (95% CI, 82.2-90.3%) (Fig. 3A). A substantial degree of heterogeneity was observed (I^2^=87.3%) (Table II). The leave-one-out sensitivity analysis further confirmed the robustness of these findings, indicating that omitting any single study did not significantly alter the pooled estimate (Fig. S2A).

*Dizziness*. Dizziness was reported in 12 studies, encompassing 749 patients, with a pooled prevalence of 30.2% (95% CI, 12.8-51.0%) (Fig. 3B). A substantial degree of heterogeneity was observed across the studies, as indicated by an I^2^ value of 96.6% (Table II). A leave-one-out sensitivity analysis confirmed that excluding individual studies at a time did not alter the overall prevalence estimate, indicating the stability of the findings (Fig. S2B).

*Meta-analyses of prevalence: Ocular manifestations*

### Eye pain

**Figure 4:**
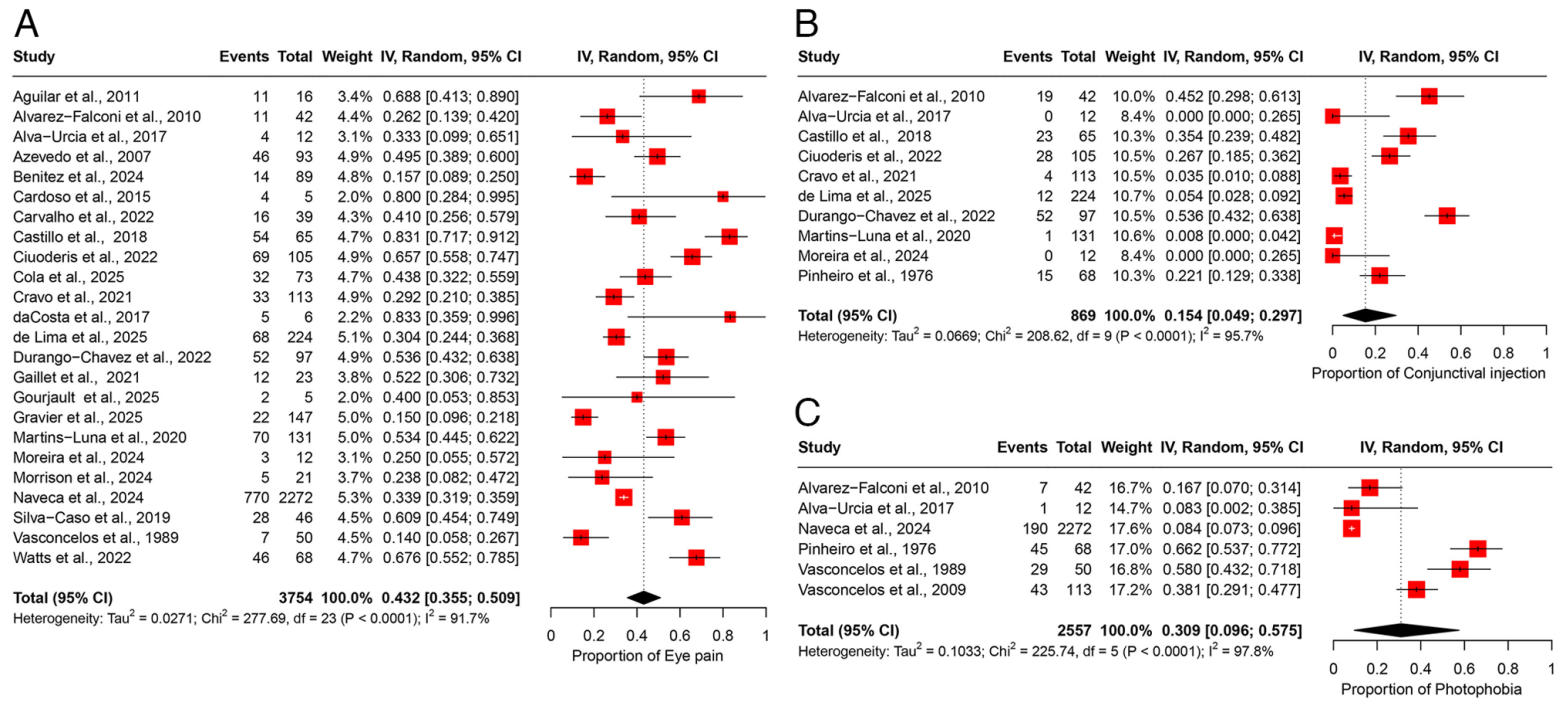
Forest plot of the prevalence of ocular manifestations among patients infected with OROV. (A) The pooled prevalence of eye pain among OROV-infected patients. (B) Prevalence of conjunctival injection among OROV-infected patients. (C) Prevalence of photophobia among OROV-infected patients. The studies included were as follows: Aguilar *et al* (24), Alvarez-Falconi *et al* (25), Alva-Urcia *et al* (26), Azevedo *et al* (11), Benitez *et al* (34), Cardoso *et al* (16), Carvalho *et al* (17), Castillo *et al* (27), Ciuoderis *et al* (36), Cola *et al* (18), Cravo *et al* (19), da Costa *et al* (20), de Lima *et al* (21), de Melo Iani *et al* (22), Durango-Chavez *et al* (28), Gaillet *et al* (32), Gourjault *et al* (33), Gravier *et al* (35), Martins-Luna *et al* (29), Moreira *et al* (2), Morrison *et al* (37), Mourão *et al* (23), Naveca *et al* (12), Pinheiro *et al* (13), Silva-Caso *et al* (30), Vasconcelos *et al* (14), Vasconcelos *et al* (15), Watts *et al* (31). OROV, Oropouche virus; 95% CI, 95% confidence interval.

A total of 24 studies comprising 3,754 participants were included in the meta-analysis of eye pain, yielding a pooled prevalence estimate of 43.2% (95% CI, 35.5-50.9%) (Fig. 4A). There was substantial heterogeneity among studies (I^2^=91.7%), suggesting considerable variability in the reported prevalence of eye pain across different study populations (Table II). Sensitivity analysis using a leave-one-out approach demonstrated that the omission of any single study had no substantial impact on the overall pooled prevalence estimate (Fig. S3A).

*Conjunctival injection*. A total of 10 studies involving 869 participants were included in the meta-analysis of conjunctival injection, yielding a pooled prevalence estimate of 15.4% (95% CI, 4.9-29.7%) (Fig. 4B). Considerable heterogeneity was observed among the included studies (I^2^=95.7%), reflecting substantial variability in reported prevalence across different populations (Table II). Sensitivity analysis using a leave-one-out approach indicated that removing any single study did not significantly influence the overall prevalence estimate (Fig. S3B).

*Photophobia*. Of note, six studies, with a total of 2,557 participants were included in the analysis of photophobia, yielding a pooled prevalence of 30.9% (95% CI, 9.6-57.5%) (Fig. 4C). Significant heterogeneity was observed across studies (I^2^=97.8%), indicating variability in prevalence estimates among different populations (Table II). Sensitivity analysis using a leave-one-out approach showed that excluding any single study did not notably affect the overall pooled estimate (Fig. S3C).

### Meta-analyses of prevalence: Respiratory manifestations

**Figure 5:**
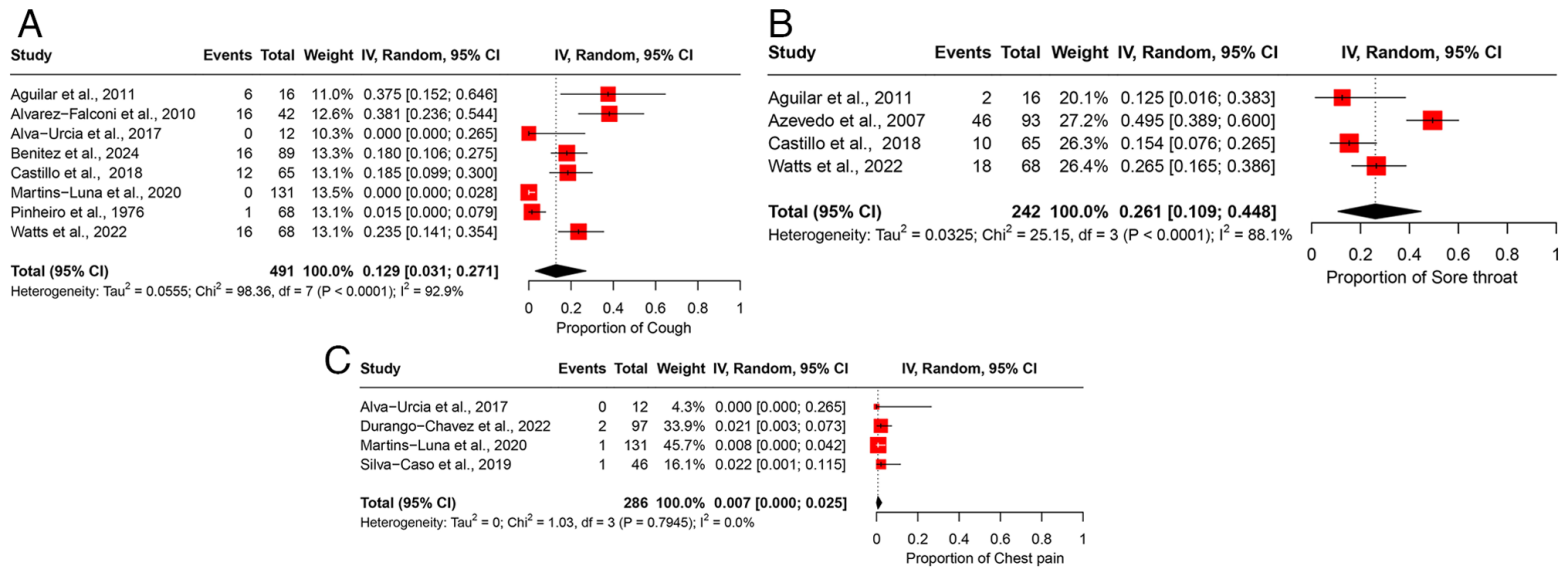
Forest plot of the prevalence of respiratory manifestations among patients infected with OROV. (A) The pooled prevalence of cough among OROV-infected patients. (B) Prevalence of sore throat among OROV-infected patients. (C) Prevalence of chest pain among OROV-infected patients. The studies included were as follows: Aguilar *et al* (24), Alvarez-Falconi *et al* (25), Alva-Urcia *et al* (26), Azevedo *et al* (11), Benitez *et al* (34), Cardoso *et al* (16), Carvalho *et al* (17), Castillo *et al* (27), Durango-Chavez *et al* (28), Martins-Luna *et al* (29), Pinheiro *et al* (13), Silva-Caso *et al* (30), Watts *et al* (31) OROV, Oropouche virus; 95% CI, 95% confidence interval.

*Cough*. A total of eight studies, comprising 491 participants, were included in the analysis of cough, resulting in a pooled prevalence of 12.9% (95% CI, 3.1-27.1%) (Fig. 5A). High heterogeneity was observed across the studies (I^2^=92.9%), indicating marked variability in the reported prevalence of cough among different study populations (Table II). Leave-one-out sensitivity analysis confirmed the stability of the pooled estimate, with the exclusion of any individual study having minimal impact on the overall result (Fig. S4A).

*Sore throat*. The analysis of sore throat included four studies with 242 participants, yielding a pooled prevalence of 26.1% (95% CI, 10.9-44.8%) (Fig. 5B). The studies exhibited high heterogeneity (I^2^=88.1%), suggesting substantial differences in the prevalence estimates across various study populations (Table II). Sensitivity analysis using the leave-one-out method indicated that removing any individual study did not notably affect the overall pooled estimate (Fig. S4B).

*Chest pain*. Four studies involving 286 participants were included in the analysis of chest pain, resulting in a pooled prevalence of 0.7% (95% CI, 0.0-2.5%) (Fig. 5C). No significant heterogeneity was observed across the studies (I^2^=0%), indicating a consistent prevalence estimate across the included populations (Table II). Leave-one-out sensitivity analysis confirmed that excluding any single study had no notable impact on the overall pooled estimate (Fig. S4C).

### Meta-analyses of prevalence: Gastrointestinal manifestations. Nausea/vomiting

**Figure 6:**
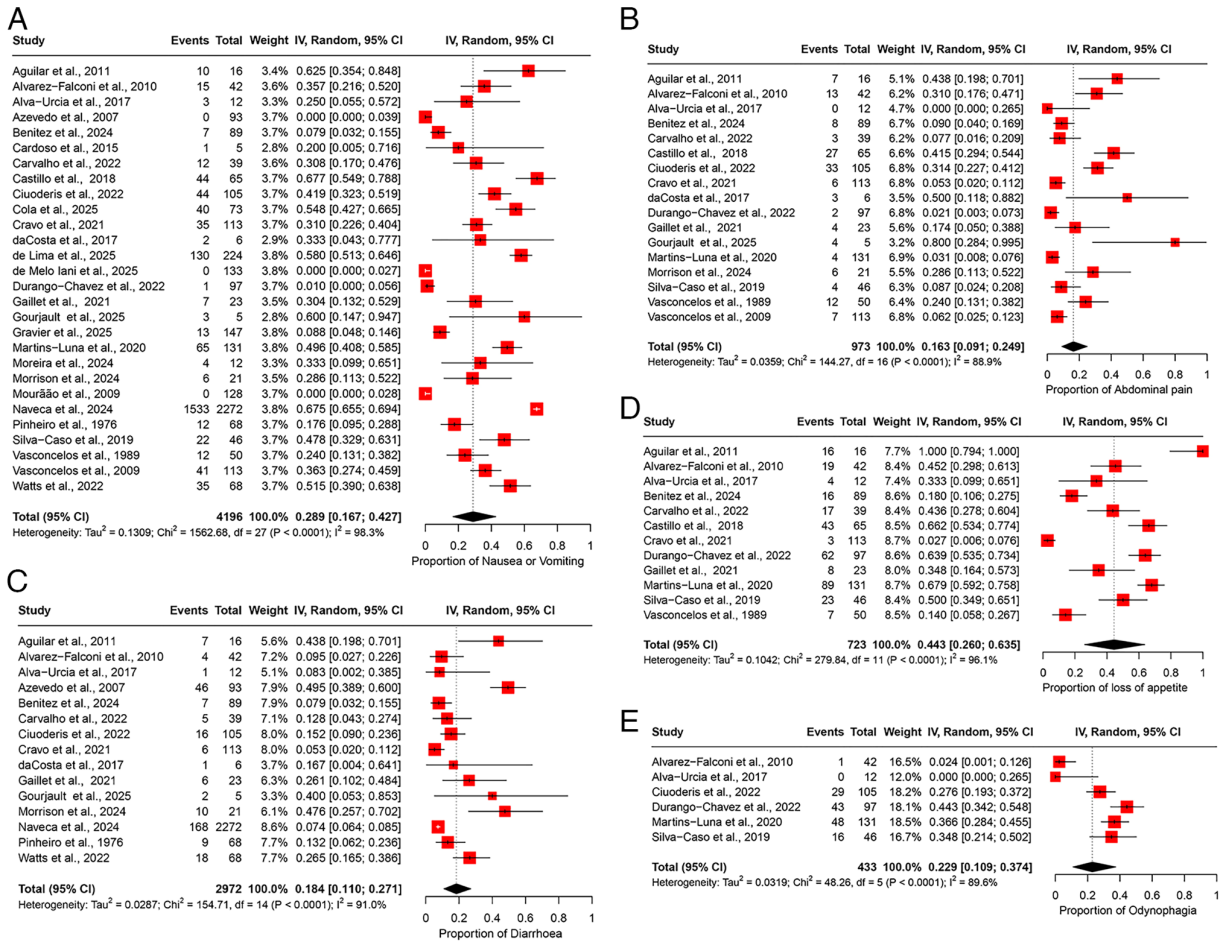
Forest plot of the prevalence of gastrointestinal manifestations among patients infected with OROV. (A) The pooled prevalence of nausea/vomiting among OROV-infected patients. (B) The pooled prevalence of abdominal pain among OROV-infected patients. (C) The pooled prevalence of diarrhea among OROV-infected patients. (D) The pooled prevalence of loss of appetite among OROV-infected patients. (E) The pooled prevalence of odynophagia among OROV-infected patients. The studies included were as follows: Aguilar *et al* (24), Alvarez-Falconi *et al* (25), Alva-Urcia *et al* (26), Azevedo *et al* (11), Benitez *et al* (34), Cardoso *et al* (16), Carvalho *et al* (17), Castillo *et al* (27), Ciuoderis *et al* (36), Cola *et al* (18), Cravo *et al* (19), da Costa *et al* (20), de Lima *et al* (21), de Melo Iani *et al* (22), Durango-Chavez *et al* (28), Gaillet *et al* (32), Gourjault *et al* (33), Gravier *et al* (35), Martins-Luna *et al* (29), Moreira *et al* (2), Morrison *et al* (37), Mourão *et al* (23), Naveca *et al* (12), Pinheiro *et al* (13), Silva-Caso *et al* (30), Vasconcelos *et al* (14), Vasconcelos *et al* (15), Watts *et al* (31) OROV, Oropouche virus; 95% CI, 95% confidence interval.

The pooled prevalence of nausea/vomiting was 28.9% (95% CI, 16.7-42.7%), based on data from 28 studies involving 4,196 patients (Fig. 6A). High heterogeneity was observed among the studies (I^2^=98.3%), indicating substantial variability in the prevalence estimates (Table II). Sensitivity analysis, performed by sequentially excluding each study, revealed that the overall prevalence estimate remained stable and was not significantly influenced by the exclusion of any individual study (Fig. S5A).

*Abdominal pain*. The pooled prevalence of abdominal pain was estimated at 16.3% (95% CI, 9.1-24.9%), based on data from 17 studies involving 973 participants (Fig. 6B). High heterogeneity was observed across the studies (I^2^=88.9%), indicating considerable variability in the prevalence estimates among different study populations (Table II). Sensitivity analysis, conducted by sequentially excluding each study at a time, confirmed that the overall prevalence estimate remained robust and was not significantly altered by excluding any individual study (Fig. S5B).

*Diarrhea*. The pooled prevalence of diarrhea was 18.4% (95% CI, 11.0-27.1%), based on data from 15 studies involving 2,972 participants (Fig. 6C), with high heterogeneity observed across the studies (I^2^=91.0%), suggesting considerable variability in the prevalence estimates among different study populations (Table II). A sensitivity analysis, excluding one study at a time, demonstrated that the overall prevalence estimate remained stable, influenced by the exclusion of any individual study (Fig. S5C).

*Loss of appetite*. The pooled prevalence of loss of appetite was 44.3% (95% CI, 26.0-63.5%), based on data from 12 studies involving 723 participants (Fig. 6D). High heterogeneity was observed among the studies (I^2^=96.1%), indicating substantial variability in the prevalence estimates across different study populations (Table II). Sensitivity analysis, performed by sequentially excluding one study at a time, revealed that the overall prevalence estimate remained stable and was not significantly affected by excluding any individual study (Fig. S5D).

*Odynophagia*. The pooled prevalence of odynophagia was 22.9% (95% CI, 10.9-37.4%), based on data from 6 studies involving 433 participants (Fig. 6E). The studies exhibited high heterogeneity (I^2^=89.6%), suggesting considerable variation in the prevalence estimates across the different study cohorts (Table II). Sensitivity analysis, conducted by excluding one study at a time, indicated that the overall prevalence estimate remained robust and was not substantially affected by the removal of any particular study (Fig. S5E).

### Meta-analyses of prevalence: Musculoskeletal manifestations. Myalgia

**Figure 7:**
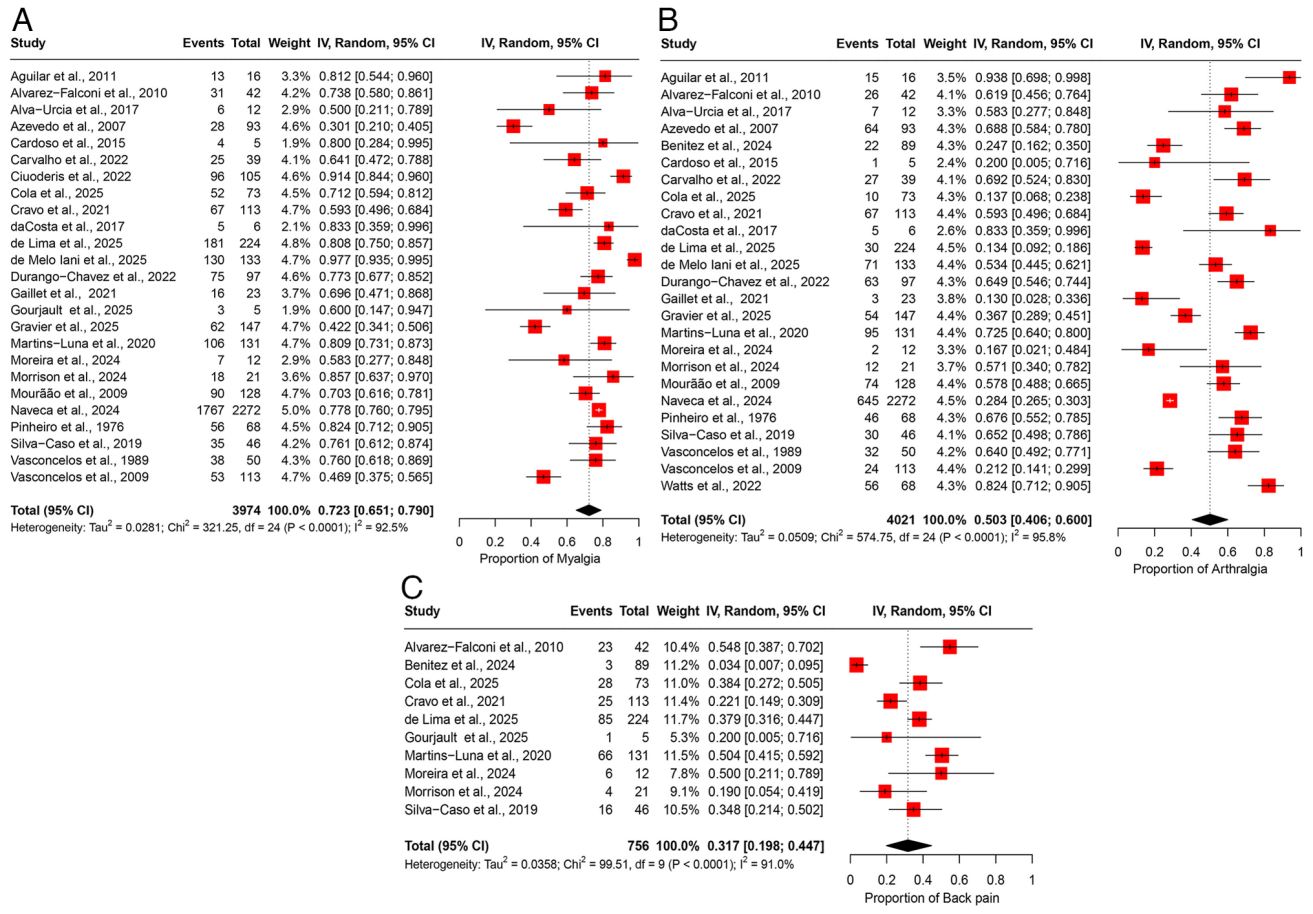
Forest plot of the prevalence of musculoskeletal manifestations among patients infected with OROV. (A) The pooled prevalence of myalgia among OROV-infected patients. (B) The pooled prevalence of arthralgia among OROV-infected patients. (C) The pooled prevalence of back pain among OROV-infected patients. The studies included were as follows: Aguilar *et al* (24), Alvarez-Falconi *et al* (25), Alva-Urcia *et al* (26), Azevedo *et al* (11), Benitez *et al* (34), Cardoso *et al* (16), Carvalho *et al* (17), Castillo *et al* (27), Ciuoderis *et al* (36), Cola *et al* (18), Cravo *et al* (19), da Costa *et al* (20), de Lima *et al* (21), de Melo Iani *et al* (22), Durango-Chavez *et al* (28), Gaillet *et al* (32), Gourjault *et al* (33), Gravier *et al* (35), Martins-Luna *et al* (29), Moreira *et al* (2), Morrison *et al* (37), Mourão *et al* (23), Naveca *et al* (12), Pinheiro *et al* (13), Silva-Caso *et al* (30), Vasconcelos *et al* (14), Vasconcelos *et al* (15), Watts *et al* (31) OROV, Oropouche virus; 95% CI, 95% confidence interval.

The pooled prevalence of myalgia was 72.3% (95% CI, 65.1-79.0%), based on data from 25 studies involving 3,974 participants (Fig. 7A). A substantial degree of heterogeneity was observed across the studies (I2=92.5%), suggesting notable variability in the prevalence estimates among the different study populations (Table II). Sensitivity analysis, conducted through a leave-one-out meta-analysis by sequentially excluding each study, demonstrated that the overall prevalence estimate remained consistent and was not significantly impacted by excluding any individual study (Fig. S6A).

*Arthralgia*. The pooled prevalence of arthralgia was 50.3% (95% CI, 40.6-60.0%), based on data from 25 studies involving 4,021 participants (Fig. 7B). Significant heterogeneity was observed across the studies (I^2^=95.8%), suggesting notable variability in the prevalence estimates across different study populations (Table II). Sensitivity analysis, conducted via a leave-one-out meta-analysis by excluding each study, revealed that the overall prevalence estimate remained stable and was not notably impacted by the exclusion of any individual study (Fig. S6B).

*Back pain*. The pooled prevalence of back pain was 31.7% (95% CI, 19.8-44.7%), based on data from 10 studies involving 756 participants (Fig. 7C). Moderate heterogeneity was observed among the studies (I^2^=91.0%), indicating substantial variability in the prevalence estimates across the study populations (Table II). Sensitivity analysis, conducted by sequentially excluding each study, demonstrated that the overall prevalence estimate remained stable and was not significantly influenced by the exclusion of any individual study (Fig. S6C).

### Meta-analyses of prevalence: Dermatological manifestations. Skin rash

**Figure 8:**
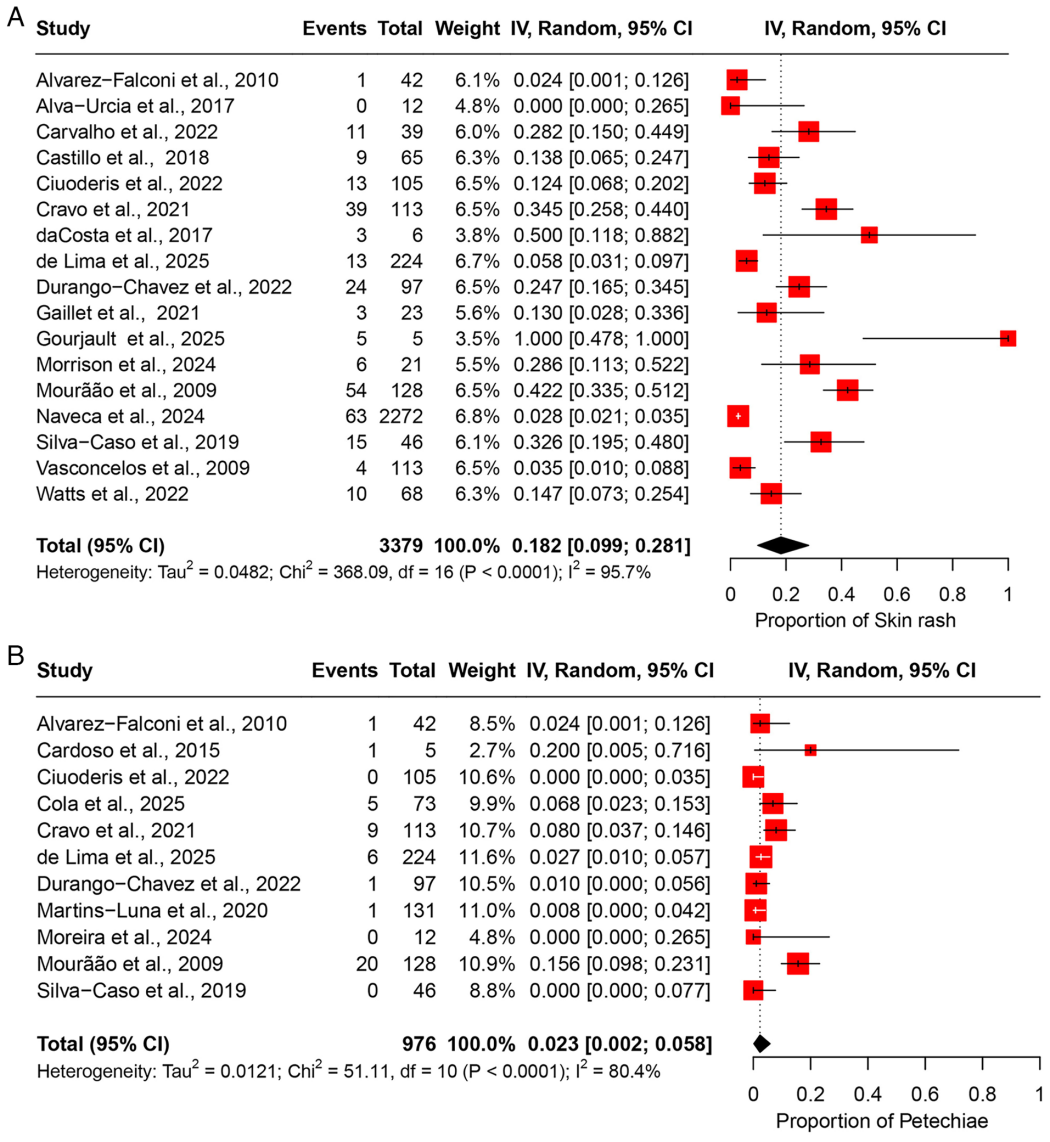
Forest plot of the prevalence of dermatological manifestations among patients infected with OROV. (A) The pooled prevalence of skin rash among OROV-infected patients. (B) The pooled prevalence of petechiae among OROV-infected patients. The studies included were as follows: Alvarez-Falconi *et al* (25), Alva-Urcia *et al* (26), Cardoso *et al* (16), Carvalho *et al* (17), Castillo *et al* (27), Ciuoderis *et al* (36), Cola *et al* (18), Cravo *et al* (19), da Costa *et al* (20), de Lima *et al* (21), Durango-Chavez *et al* (28), Gaillet *et al* (32), Gourjault *et al* (33), Martins-Luna *et al* (29), Moreira *et al* (2), Morrison *et al* (37), Mourão *et al* (23), Naveca *et al* (12), Silva-Caso *et al* (30), Vasconcelos *et al* (14), Watts *et al* (31). OROV, Oropouche virus; 95% CI, 95% confidence interval.

The pooled prevalence of skin rash was 18.2% (95% CI, 9.9-28.1%), based on data from 17 studies involving 3,379 participants (Fig. 8A). High heterogeneity was observed across the studies (I^2^=95.7%), indicating substantial variability in the prevalence estimates among the study populations (Table II). Sensitivity analysis, performed by sequentially excluding each study, revealed that the overall prevalence estimate remained stable and was not significantly influenced by the exclusion of any individual study (Fig. S7A).

*Petechiae*. Based on 11 studies comprising 976 participants, the pooled prevalence of petechiae was estimated at 2.3% (95% CI, 0.2-5.8%) (Fig. 8B). The analysis revealed moderate heterogeneity (I^2^=80.4%), indicating certain inconsistency in prevalence rates across the included studies (Table II). A leave-one-out sensitivity analysis showed that the pooled estimate remained consistent, with no single study exerting a disproportionate influence on the overall result (Fig. S7B).

### Publication bias

Funnel plots were generated for each of the clinical symptoms assessed to evaluate the presence of publication bias. All plots appeared symmetrical upon visual inspection, suggesting no apparent publication bias across the included studies. A representative funnel plot for fever is presented in Fig. S8 to avoid redundancy. Furthermore, Begg's rank correlation tests were conducted for each outcome and yielded non-significant results (P>0.05), providing additional evidence against the presence of publication bias.

## Discussion

*Findings of the present study.* To the best of our knowledge, the present study is the largest and most comprehensive to date in examining all clinical presentations resulting from OROV infection through a systematic review and meta-analysis of cases reported from six countries across the globe. Common clinical manifestations with a prevalence >60% included fever (97%), headache (86.5%) and myalgia (72.3%). Manifestations with a prevalence between 20 and 60% were defined as intermediate presentations, including malaise or fatigue (56.4%), arthralgia (50.3%), chills (49.6%), loss of appetite (44.3%), eye pain (43.2%), back pain (31.7%), pallor (31.7%), dizziness (30.2%), photophobia (30.9%), nausea/vomiting (28.9%), sore throat (26.1%) and odynophagia (22.9%). Manifestations with a prevalence <20% were defined as rare presentations, including diarrhea (18.4%), skin rash (18.2%), conjunctival injection (15.4%), abdominal pain (16.3%), petechiae (2.3%), cough (12.9%) and chest pain (0.7%). The findings of the present study provide a valuable reference for frontline clinicians in OROV-endemic regions, emphasizing that the presence of fever, headache and myalgia, primarily when occurring together, should raise clinical suspicion for OROV infection. The early recognition of intermediate and rare manifestations can further support timely diagnosis, inform differential diagnoses, and improve case management and patient outcomes during outbreaks.

*Comparison with previous studies*. Studies on OROV infection outcomes with large sample sizes are limited, with the most extensive study to date being conducted by Naveca *et al* in Brazil (12), which included 2,272 patients. The majority of existing literature consists of case reports or small cohort studies, leaving the clinical presentation of OROV unclear. To address this issue, the present systematic review and meta-analysis was conducted in an aim to provide a better understanding of the symptom profile of OROV based on previously published studies. Despite being recognized as an emerging public health threat in Latin America, OROV remains understudied compared to other arboviruses, such as dengue or Zika. Notably, while only a small number of systematic reviews and meta-analyses on OROV have been published (1,38,39), the majority focus on narrow aspects of the virus, such as its epidemiology or specific outbreaks, and often include smaller cohorts than the present study. The limited scope of these studies has left critical gaps in the understanding of the full clinical spectrum, transmission dynamics and global burden of OROV. The present meta-analysis included studies from six countries, with the majority of data derived from Brazil (2,11-23) and Peru (24-31), both highly endemic regions for OROV. Across these regions, fever, headache and myalgia consistently emerged as the most prevalent symptoms. However, certain manifestations, such as rash, odynophagia and gastrointestinal symptoms, exhibited variations in prevalence between countries. For instance, rash was reported more frequently in Brazilian cohorts (17) compared to those from Peru (27), potentially reflecting differences in viral genotypes, vector ecology, or case ascertainment practices. Similarly, gastrointestinal symptoms, such as nausea and diarrhea were more commonly observed in Peruvian studies (24-31). These regional differences underscore the importance of considering geographic context when diagnosing and managing OROV infection. Variability in healthcare access, surveillance systems and diagnostic criteria may also influence reported symptom profiles. Understanding these variations is critical for tailoring public health responses and clinical management strategies in distinct endemic areas.

The findings presented herein align with the previous meta-analysis performed by Wang *et al* (38), which pooled data from 15 studies involving 806 OROV-infected patients. Their analysis identified fever, headache, myalgia and arthralgia as the most common clinical manifestations of OROV infection. However, that meta-analysis did not include the most extensive study on OROV infection, conducted by Naveca *et al* (12), which analyzed data from 2,272 patients, limiting its generalizability. By contrast, the present study integrated data from 28 studies involving 4,196 participants, which is >5-fold greater than the sample size of the previous meta-analysis (38), providing a broader and more representative symptom profile. This expanded analysis allows for greater precision in estimating symptom prevalence and enhances the understanding of the clinical presentation of OROV, which is critical for timely diagnosis, appropriate case management and targeted public health interventions in endemic regions.

Moreover, the findings of the present study are consistent with those in the study by Tortosa *et al* (39), particularly in the high prevalence of fever and headache in OROV, which were consistently reported in both studies. Both analyses also demonstrate a notable association with musculoskeletal symptoms, such as myalgia and arthralgia. However, the present meta-analysis broadens the scope of the study by Tortosa *et al* (39) by including additional symptoms, such as chills, malaise and back pain, which were less frequently observed in their research. Additionally, while both studies report gastrointestinal symptoms such as nausea and diarrhea, the results presented herein demonstrate a higher prevalence, potentially indicating regional variations. These results reinforce the need for comprehensive symptom surveillance to ensure accurate diagnosis and effective monitoring. The systematic review by Pereira *et al* (40) examined the epidemiological distribution and exposure rates of OROV in South America, whereas the present meta-analysis emphasized the clinical manifestations of OROV infection, providing a more in-depth understanding of its clinical features and aiding in the development of better diagnostic and public health approaches in areas where the virus is endemic. The systematic review and meta-analysis performed by Kharwadkar and Herath (41) examined the clinical manifestations of dengue, Zika and chikungunya in the Pacific Islands, revealing distinct symptom profiles among these arboviruses. Their study found that dengue was most frequently associated with fever and headache, whereas a higher prevalence of rash, conjunctivitis and peripheral edema characterized Zika infections (41). Chikungunya, by contrast, was predominantly associated with fever and arthralgia. Additionally, the study by Kharwadkar and Herath (41) highlighted the occurrence of Guillain-Barré syndrome (GBS) in both Zika and chikungunya, with dengue having a hospitalization rate of 9.90% and a mortality rate of 0.23%. When comparing these findings to our study on OROV, significant differences emerge. OROV demonstrates a higher prevalence of ocular manifestations, such as conjunctival injection and photophobia, which were not as prominent in the other arboviruses discussed Kharwadkar and Herath (41). Furthermore, neurological symptoms, including dizziness, were more commonly observed in OROV infections, distinguishing it from the less frequent neurological manifestations in dengue, Zika and chikungunya. The unique symptom profile, particularly the ocular and neurological features, highlights the distinct clinical presentation of OROV compared to the more commonly shared manifestations of fever, headache and rash in other arboviral infections.

The present systematic review and meta-analysis aimed to address the limitations of previous studies by providing a more comprehensive and methodologically rigorous synthesis of the clinical manifestations associated with OROV infection. The substantial heterogeneity observed across many clinical manifestations (I^2^>75%) suggests significant variability in symptom reporting across the included studies. This variability may stem from differences in diagnostic criteria, healthcare access, viral strain diversity, surveillance intensity and population characteristics. From a clinical standpoint, this poses challenges for accurate diagnosis and public health monitoring, particularly in regions where OROV co-circulates with other arboviruses such as dengue or Zika. Inconsistent symptom definitions across studies may also contribute to reporting bias, particularly for less common or subjective symptoms. These findings underscore the urgent need for internationally standardized clinical definitions and classification criteria for OROV infection, particularly for identifying severe or complicated cases. The elevated prevalence of clinical symptoms identified in the present analysis may, in part, be attributed to the inclusion of 14 studies from Brazil and eight from Peru, countries with high endemicity of OROV. Although the present study incorporated data from six countries, the predominance of studies originating from Brazil and Peru may constrain the generalizability of the findings to other geographic regions. These two countries not only report higher levels of OROV endemicity, but also benefit from relatively advanced surveillance infrastructure, potentially influencing the detection and characterization of clinical manifestations. Variations in circulating OROV genotypes, diagnostic capacity and co-infection rates in other parts of Latin America, particuarlly in regions beyond the continent, may yield different clinical presentations. Accordingly, caution is warranted when extrapolating these results to underrepresented or non-endemic areas. Additional research encompassing a more diverse geographic locations is essential to validate and extend these findings.

The present study has several notable strengths. First, the present meta-analysis evaluated the system-wise clinical manifestations of OROV infection. Understanding these manifestations can help identify the most affected systems, allowing healthcare professionals to prioritize diagnostic and therapeutic strategies that may enhance patient management and deepen our understanding of OROV pathophysiology. Second, a sensitivity analyses was conducted for all clinical manifestations of OROV infection, which were not addressed in previous meta-analyses. This approach enhances the robustness and validity of the findings. Finally, a key strength of the present study is its global scope and large-scale meta-analysis, which includes data from six countries. Unlike previous analyses, which mainly relied on case reports and studies with small sample sizes, our study incorporated 28 studies with over 4,100 cases of OROV infection, offering a more comprehensive and robust evaluation.

However, the present study has several limitations, which should be mentioned. First, the majority of studies included in the present meta-analysis were retrospective cohort studies or case series, with limited data from prospective designs, which may introduce inherent selection and reporting biases and potentially affect the robustness of the findings. Second, since the majority of studies included in the present meta-analysis were conducted in South America, the applicability of the results to global populations may be constrained. Thirdly, significant heterogeneity was noted in the pooled analysis of several clinical manifestations of OROV infection. The differences in findings across various study locations imply that variations in reporting standards across countries may have played a significant role in the observed heterogeneity. Finally, the authors were unable to construct a phylogenetic tree of OROV strains, as genomic sequence data were not consistently reported in the included studies. This limits the ability to explore the genetic diversity and evolutionary dynamics of OROV

In conclusion, to the best of our knowledge, the present systematic review and meta-analysis is the first and most comprehensive study examining the full spectrum of clinical manifestations of OROV infection, analyzing cases from six countries. The most commonly reported manifestations of OROV were fever, headache, malaise or fatigue, chills, eye pain, etc. This study contributes significantly to understanding the current OROV outbreak, providing valuable insight for future research into the pathological mechanisms and epidemiology of the disease. Through a meta-analysis of clinical manifestations of OROV infection, the present study provides a comprehensive understanding of its symptoms, aiding in early diagnosis and improving diagnostic accuracy. It highlights symptom variability across populations, informs public health strategies, and guides future research into the pathophysiology and epidemiology of OROV. However, future research is required to prioritize longitudinal studies to investigate symptom progression, explore additional OROV manifestations, and extend geographic analysis to improve the generalizability of the findings.

## Supplementary Material

Sensitivity analysis of included studies for general or systemic manifestations. (A) Sensitivity analysis of included studies for fever. (B) Sensitivity Analysis of Included studies for malaise or fatigue. (C) Sensitivity analysis of Included studies for chills. (D) Sensitivity analysis of included studies for pallor. The studies included were as follows: Aguilar *et al* (24), Alvarez-Falconi *et al* (25), Alva-Urcia *et al* (26), Azevedo *et al* (11), Benitez *et al* (34), Cardoso *et al* (16), Carvalho *et al* (17), Castillo *et al* (27), Ciuoderis *et al* (36), Cola *et al* (18), Cravo *et al* (19), da Costa *et al* (20), de Lima *et al* (21), de Melo Iani *et al* (22), Durango-Chavez *et al* (28), Gaillet *et al* (32), Gourjault *et al* (33), Gravier *et al* (35), Martins-Luna *et al* (29), Moreira *et al* (2), Morrison *et al* (37), Mourão *et al* (23), Naveca *et al* (12), Pinheiro *et al* (13), Silva-Caso *et al* (30), Vasconcelos *et al* (14), Vasconcelos *et al* (15), Watts *et al* (31) 95% CI, 95% confidence interval.

Sensitivity analysis of included studies for neurological manifestations. (A) Sensitivity analysis of included studies for headache. (B) Sensitivity analysis of included studies for dizziness. The studies included were as follows: Aguilar *et al* (24), Alvarez-Falconi *et al* (25), Alva-Urcia *et al* (26), Azevedo *et al* (11), Benitez *et al* (34), Cardoso *et al* (16), Carvalho *et al* (17), Castillo *et al* (27), Ciuoderis *et al* (36), Cola *et al* (18), Cravo *et al* (19), da Costa *et al* (20), de Lima *et al* (21), de Melo Iani *et al* (22), Durango-Chavez *et al* (28), Gaillet *et al* (32), Gourjault *et al* (33), Gravier *et al* (35), Martins-Luna *et al* (29), Moreira *et al* (2), Morrison *et al* (37), Mourão *et al* (23), Naveca *et al* (12), Pinheiro *et al* (13), Silva-Caso *et al* (30), Vasconcelos *et al* (14), Vasconcelos *et al* (15), Watts *et al* (31) 95% CI, 95% confidence interval.

Sensitivity analysis of included studies for ocular manifestations. (A) Sensitivity analysis of included studies for eye pain. (B) Sensitivity analysis of included studies for conjunctival injection. (C) Sensitivity analysis of included studies for photophobia. The studies included were as follows: Aguilar *et al* (24), Alvarez-Falconi *et al* (25), Alva-Urcia *et al* (26), Azevedo *et al* (11), Benitez *et al* (34), Cardoso *et al* (16), Carvalho *et al* (17), Castillo *et al* (27), Ciuoderis *et al* (36), Cola *et al* (18), Cravo *et al* (19), da Costa *et al* (20), de Lima *et al* (21), de Melo Iani *et al* (22), Durango-Chavez *et al* (28), Gaillet *et al* (32), Gourjault *et al* (33), Gravier *et al* (35), Martins-Luna *et al* (29), Moreira *et al* (2), Morrison *et al* (37), Mourão *et al* (23), Naveca *et al* (12), Pinheiro *et al* (13), Silva-Caso *et al* (30), Vasconcelos *et al* (14), Vasconcelos *et al* (15), Watts *et al* (31) 95% CI, 95% confidence interval.

Sensitivity analysis of included studies for respiratory manifestations. (A) Sensitivity analysis of included studies for cough. (B) Sensitivity analysis of included studies for sore throat. (C) Sensitivity analysis of included studies for chest pain. The studies included were as follows: Aguilar *et al* (24), Alvarez-Falconi *et al* (25), Alva-Urcia *et al* (26), Azevedo *et al* (11), Benitez *et al* (34), Cardoso *et al* (16), Carvalho *et al* (17), Castillo *et al* (27), Durango-Chavez *et al* (28), Martins-Luna *et al* (29), Pinheiro *et al* (13), Silva-Caso *et al* (30), Watts *et al* (31) 95% CI, 95% confidence interval.

Sensitivity analysis of included studies for gastrointestinal manifestations. (A) Sensitivity analysis of Included studies for nausea or vomiting. (B) Sensitivity analysis of included studies for abdominal pain. (C) Sensitivity analysis of included studies for diarrhoea. (D) Sensitivity analysis of included studies for loss of appetite. (E) sensitivity analysis of included studies for odynophagia. The studies included were as follows: Aguilar *et al* (24), Alvarez-Falconi *et al* (25), Alva-Urcia *et al* (26), Azevedo *et al* (11), Benitez *et al* (34), Cardoso *et al* (16), Carvalho *et al* (17), Castillo *et al* (27), Ciuoderis *et al* (36), Cola *et al* (18), Cravo *et al* (19), da Costa *et al* (20), de Lima *et al* (21), de Melo Iani *et al* (22), Durango-Chavez *et al* (28), Gaillet *et al* (32), Gourjault *et al* (33), Gravier *et al* (35), Martins-Luna *et al* (29), Moreira *et al* (2), Morrison *et al* (37), Mourão *et al* (23), Naveca *et al* (12), Pinheiro *et al* (13), Silva-Caso *et al* (30), Vasconcelos *et al* (14), Vasconcelos *et al* (15), Watts *et al* (31). 95% CI, 95% confidence interval.

Sensitivity analysis of included studies for musculoskeletal manifestations. (A) Sensitivity analysis of included studies for myalgia. (B) Sensitivity analysis of included studies for arthralgia. (C) Sensitivity analysis of included studies for back pain. The studies included were as follows: Aguilar *et al* (24), Alvarez-Falconi *et al* (25), Alva-Urcia *et al* (26), Azevedo *et al* (11), Benitez *et al* (34), Cardoso *et al* (16), Carvalho *et al* (17), Castillo *et al* (27), Ciuoderis *et al* (36), Cola *et al* (18), Cravo *et al* (19), da Costa *et al* (20), de Lima *et al* (21), de Melo Iani *et al* (22), Durango-Chavez *et al* (28), Gaillet *et al* (32), Gourjault *et al* (33), Gravier *et al* (35), Martins-Luna *et al* (29), Moreira *et al* (2), Morrison *et al* (37), Mourão *et al* (23), Naveca *et al* (12), Pinheiro *et al* (13), Silva-Caso *et al* (30), Vasconcelos *et al* (14), Vasconcelos *et al* (15), Watts *et al* (31) 95% CI, 95% confidence interval.

Sensitivity analysis of included studies for dermatological manifestations. (A) Sensitivity analysis of included studies for skin rash. (B) Sensitivity analysis of included studies for petechiae. The studies included were as follows: Alvarez-Falconi *et al* (25), Alva-Urcia *et al* (26), Cardoso *et al* (16), Carvalho *et al* (17), Castillo *et al* (27), Ciuoderis *et al* (36), Cola *et al* (18), Cravo *et al* (19), da Costa *et al* (20), de Lima *et al* (21), Durango-Chavez *et al* (28), Gaillet *et al* (32), Gourjault *et al* (33), Martins-Luna *et al* (29), Moreira *et al* (2), Morrison *et al* (37), Mourão *et al* (23), Naveca *et al* (12), Silva-Caso *et al* (30), Vasconcelos *et al* (14), Watts *et al* (31) 95% CI, 95% confidence interval.

Funnel plot assessing publication bias for fever among the included studies.

The detailed search strategy for each database.

Quality appraisal of studies using the modified Newcastle-Ottawa Scale.

Methodological quality assessment of case series/reports using the tool described in the study by Murad *et al* (10)^a^.

## Figures and Tables

**Table I:** Characteristics of the OROV-infected patients in the included studies.

First author, year of publication	Country	Total no. of patients	Study type	Study period	Test method	NOS Score	(Refs.)
Aguilar, 2011	Peru	16	Cross-sectional	1999 to 2006	Virus isolation and serological assay (ELISA)	5	(24)
Alvarez-Falconi, 2010	Peru	42	Cross sectional	2010	Serology (ELISA IgM)	5	(25)
Alva-Urcia, 2017	Peru	12	Cross-sectional	January 2016 to March 2016	Conventional PCR	5	(26)
Azevedo, 2007	Brazil	93	Cross-sectional	2003 to 2004	Hemagglutination inhibition, ELISA	5	(11)
Benitez, 2024	Cuba	89	Cross-sectional	2024	RT-PCR	5	(34)
Cardoso, 2015	Brazil	5	Cross-sectional	2011 to 2012	RT-PCR	5	(16)
Carvalho, 2022	Brazil	39	Cross-sectional	2018	Virus isolation, ELISA	5	(17)
Castillo, 2018	Peru	65	Cohort study	1995 to 2013	Virus culture, RT-PCR	5	(27)
Ciuoderis, 2022	Colombia	105	Cross-sectional	February 2019 to January 2022	RT-PCR	5	(36)
Cola, 2025	Brazil	73	Case series	March to December 2024	RT-PCR	-	(18)
Cravo, 2021	Brazil	113	Cohort study	2010 to 2019	RT-PCR	5	(19)
Da Costa, 2017	Brazil	6	Cross-sectional	2011 to 2013	ELISA	5	(20)
de Lima, 2025	Brazil	224	Cohort study	January to December 2024	RT-PCR	5	(21)
de Melo Iani, 2025	Brazil	133	Cross-sectional	2022 to 2024	RT-PCR	5	(22)
Durango-Chavez, 2022	Peru	97	Cross-sectional	January 2015 to December 2016	Molecular RT-PCR test	5	(28)
Gaillet, 2021	France	23	Cross-sectional	2020	RT-PCR test	5	(32)
Gourjault, 2025	France	5	Case series	July to August 2024	RT-PCR test, Virus isolation, ELISA	-	(33)
Gravier, 2025	Cuba	147	Cross-sectional	May to July 2024	RT-PCR test	5	(35)
Martins-Luna, 2020	Peru	131	Cross-sectional	Feburary 2016 to September 2016	Molecular RT-PCR test	5	(29)
Moreira, 2024	Brazil	12	Cross-sectional	January 2022 to March 2023	RT-PCR test	5	(2)
Morrison, 2024	USA	21	Cross-sectional	2023 to 2024	RT-PCR test	5	(37)
Mourãão, 2009	Brazil	128	Cross-sectional	2007 to 2008	ELISA	5	(23)
Naveca, 2024	Brazil	2,272	Cross-sectional	August 2022 to Feburary 2024	Duplex RT-qPCR	6	(12)
Pinheiro, 1976	Brazil	68	Cross-sectional	1976	Virus isolation, Serology	4	(13)
Silva-Caso, 2019	Peru	46	Cross-sectional	January 2016 to July 2016	Molecular RT-PCR test	5	(30)
Vasconcelos, 1989	Brazil	50	Cross-sectional	1988	Virus isolation, ELISA	4	(14)
Vasconcelos, 2009	Brazil	113	Cross-sectional	May 2006 to June 2006	Serology, hemagglutination inhibition (HI), Virus isolation, RT-PCR	4	(15)
Watts, 2022	Peru	68	Cross-sectional	October 1993 to September 1999	Virus isolation, ELISA	5	(31)

NOS, Newcastle-Ottawa Scale; RT-PCR, reverse transcription-polymerase chain reaction; ELISA, enzyme-linked immunosorbent assay; OROV, Oropouche virus.

**Table II:** Meta-analysis of clinical manifestations in patients infected with OROV.

Variable	No. of studies	Prevalence (%)	95% CI	No. of patients	I^2^^a^	T^2^^b^	P-value
General or systemic symptoms							
Fever	28	97.0	93.5-99.4	4,196	93.1	0.0312	<0.001
Chills	14	49.6	27.0-72.2	950	98.0	0.1842	<0.001
Malaise or fatigue	14	56.4	36.6-75.2	726	95.9	0.1167	<0.001
Pallor	3	31.7	21.5-42.7	93	6.5	0.0008	0.343
Neurological symptoms							
Headache	28	86.5	82.2-90.3	4,196	87.3	0.0159	<0.001
Dizziness	12	30.2	12.8-51.0	749	96.6	0.1182	<0.001
Occular manifestations							
Eye pain	24	43.2	35.5-50.9	3,754	91.7	0.0271	<0.001
Conjunctival injection	10	15.4	4.9-29.7	869	95.7	0.0669	<0.001
Photophobia	6	30.9	9.6-57.5	2,557	97.8	0.1033	<0.001
Respiratory manifestations							
Cough	8	12.9	3.1-27.1	491	92.9	0.0555	<0.001
Sore throat	4	26.1	10.9-44.8	242	88.1	0.0325	<0.001
Chest pain	4	0.7	0.0-2.5	286	0	0	0.795
Gastrointestinal symptoms							
Nausea/vomiting	28	28.9	16.7-42.7	4,196	98.3	0.1309	<0.001
Abdominal pain	17	16.3	9.1-24.9	973	88.9	0.0359	<0.001
Diarrhea	15	18.4	11.0-27.1	2,972	91.0	0.0287	<0.001
Loss of appetite	12	44.3	26.0-63.5	723	96.1	0.1042	<0.001
Odynophagia	6	22.9	10.9-37.4	433	89.6	0.0319	<0.001
Musculoskeletal manifestations							
Myalgia	25	72.3	65.1-79.0	3,974	92.5	0.0281	<0.001
Arthralgia	25	50.3	40.6-60.0	4,021	95.8	0.0509	<0.001
Back pain	10	31.7	19.8-44.7	756	91.0	0.0358	<0.001
Dermatological manifestations							
Skin rash	17	18.2	9.9-28.1	3,379	95.7	0.0482	<0.001
Petechiae	11	2.3	0.2-5.8	976	80.4	0.0121	<0.001

^a^I^2^index to quantify the degree of heterogeneity.

^b^Tau-squared as a measure of heterogeneity. 95% CI, 95% confidence interval; OROV, Oropouche virus.

## Data Availability

The data generated in the present study may be requested from the corresponding author.
