# Supplementary material for: Clinical manifestations of Oropouche virus infection: A systematic review and meta-analysis
Source: Med Int (Lond). 2025 Aug 27;5(6):67. doi: 10.3892/mi.2025.266 (PMC12416135; doi:10.3892/mi.2025.266)

Figure S1. Sensitivity analysis of included studies for general or systemic manifestations. (A) Sensitivity analysis of included studies for fever. (B) Sensitivity Analysis of Included studies for malaise or fatigue. (C) Sensitivity analysis of Included studies for chills. (D) Sensitivity analysis of included studies for pallor. The studies included were as follows: Aguilar *et al* (24), Alvarez-Falconi *et al* (25), Alva-Urcia *et al* (26), Azevedo *et al* (11), Benitez *et al* (34), Cardoso *et al* (16), Carvalho *et al* (17), Castillo *et al* (27), Ciuoderis *et al* (36), Cola *et al* (18), Cravo *et al* (19), da Costa *et al* (20), de Lima *et al* (21), de Melo Iani *et al* (22), Durango-Chavez *et al* (28), Gaillet *et al* (32), Gourjault *et al* (33), Gravier *et al* (35), Martins-Luna *et al* (29), Moreira *et al* (2), Morrison *et al* (37), Mourão *et al* (23), Naveca *et al* (12), Pinheiro *et al* (13), Silva-Caso *et al* (30), Vasconcelos *et al* (14), Vasconcelos *et al* (15), Watts *et al* (31) 95% CI, 95% confidence interval.

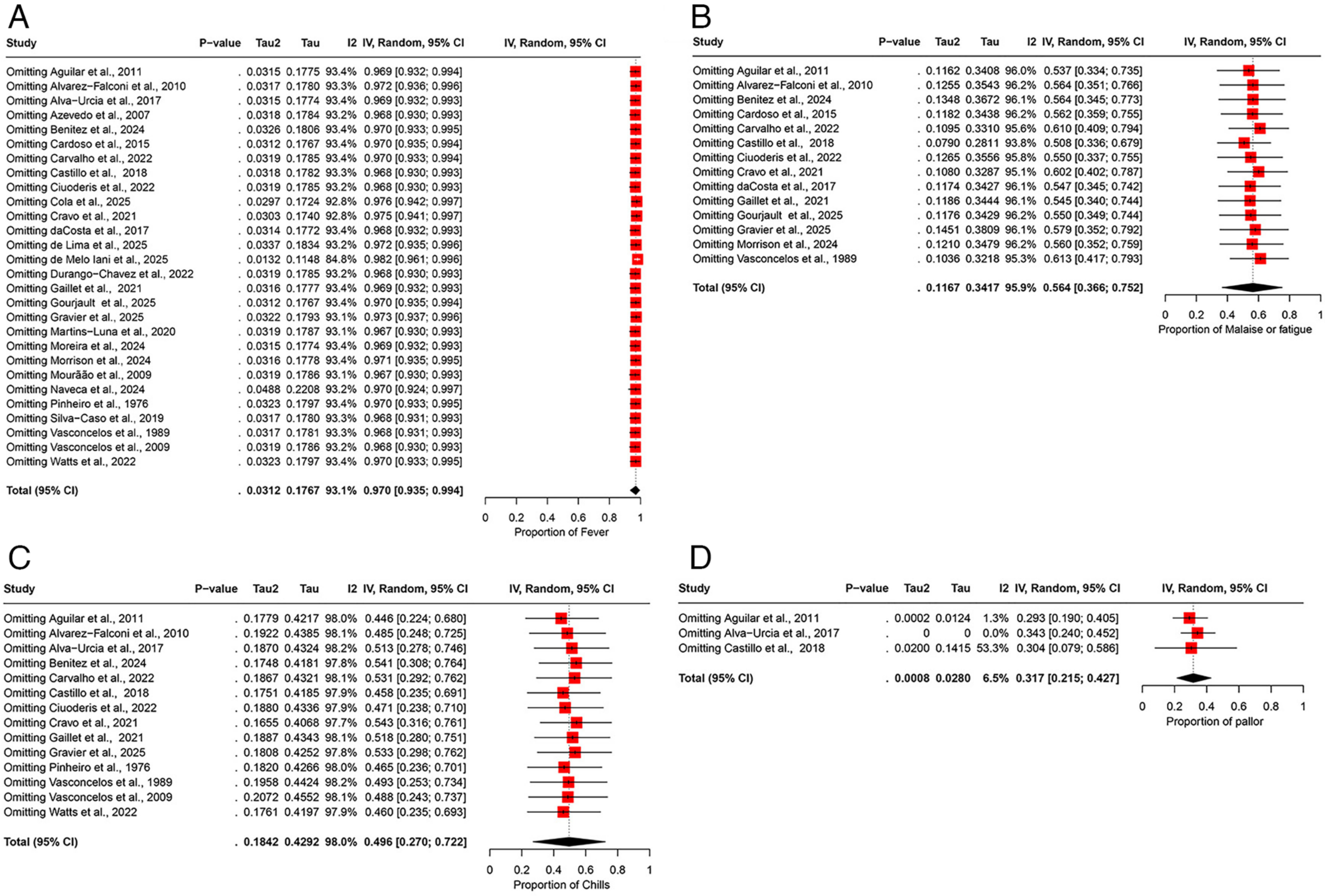

Figure S2. Sensitivity analysis of included studies for neurological manifestations. (A) Sensitivity analysis of included studies for headache. (B) Sensitivity analysis of included studies for dizziness. The studies included were as follows: Aguilar *et al* (24), Alvarez-Falconi *et al* (25), Alva-Urcia *et al* (26), Azevedo *et al* (11), Benitez *et al* (34), Cardoso *et al* (16), Carvalho *et al* (17), Castillo *et al* (27), Ciuoderis *et al* (36), Cola *et al* (18), Cravo *et al* (19), da Costa *et al* (20), de Lima *et al* (21), de Melo Iani *et al* (22), Durango-Chavez *et al* (28), Gaillet *et al* (32), Gourjault *et al* (33), Gravier *et al* (35), Martins-Luna *et al* (29), Moreira *et al* (2), Morrison *et al* (37), Mourão *et al* (23), Naveca *et al* (12), Pinheiro *et al* (13), Silva-Caso *et al* (30), Vasconcelos *et al* (14), Vasconcelos *et al* (15), Watts *et al* (31) 95% CI, 95% confidence interval.

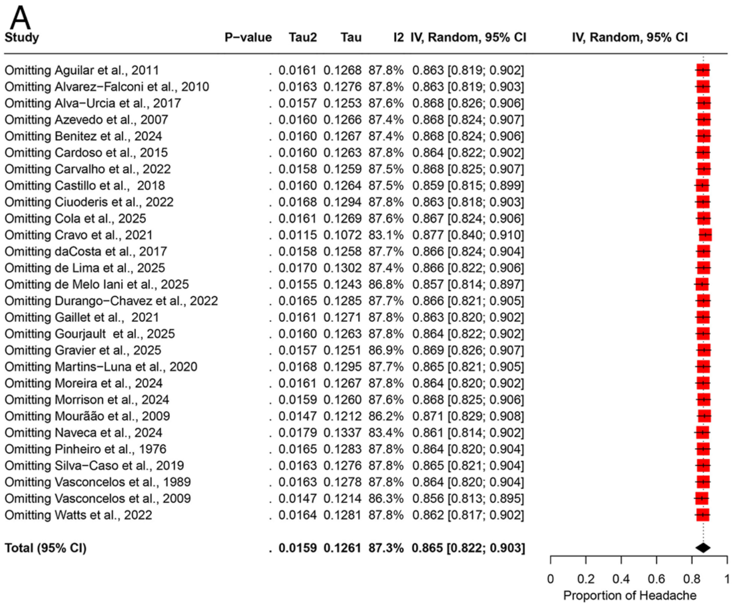

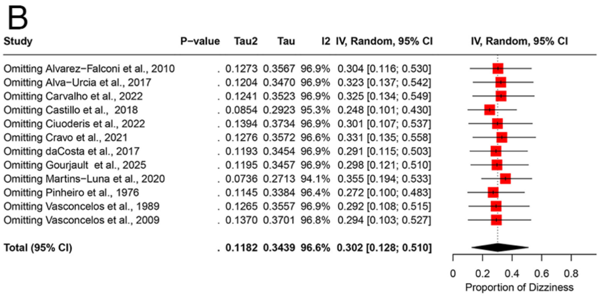

Figure S3. Sensitivity analysis of included studies for ocular manifestations. (A) Sensitivity analysis of included studies for eye pain. (B) Sensitivity analysis of included studies for conjunctival injection. (C) Sensitivity analysis of included studies for photophobia. The studies included were as follows: Aguilar *et al* (24), Alvarez-Falconi *et al* (25), Alva-Urcia *et al* (26), Azevedo *et al* (11), Benitez *et al* (34), Cardoso *et al* (16), Carvalho *et al* (17), Castillo *et al* (27), Ciuoderis *et al* (36), Cola *et al* (18), Cravo *et al* (19), da Costa *et al* (20), de Lima *et al* (21), de Melo Iani *et al* (22), Durango-Chavez *et al* (28), Gaillet *et al* (32), Gourjault *et al* (33), Gravier *et al* (35), Martins-Luna *et al* (29), Moreira *et al* (2), Morrison *et al* (37), Mourão *et al* (23), Naveca *et al* (12), Pinheiro *et al* (13), Silva-Caso *et al* (30), Vasconcelos *et al* (14), Vasconcelos *et al* (15), Watts *et al* (31) 95% CI, 95% confidence interval.

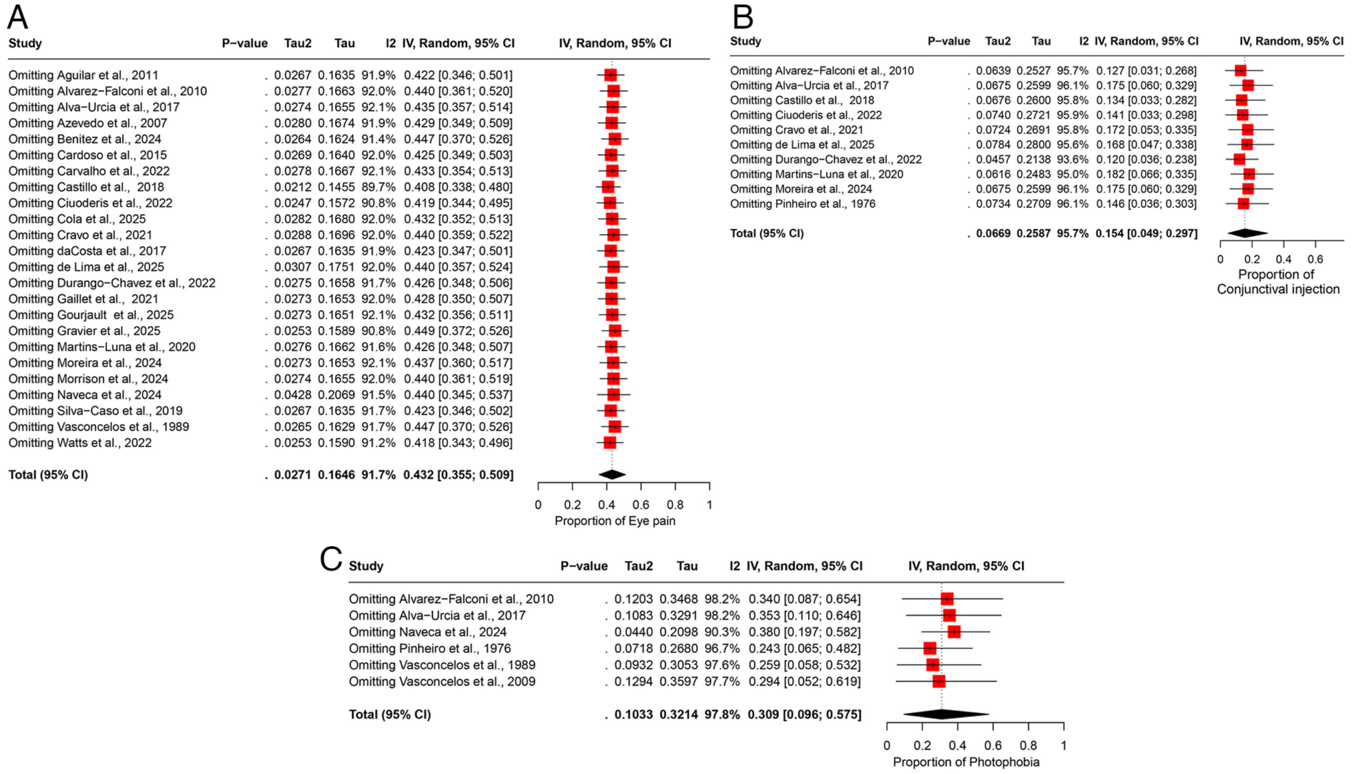

Figure S4. Sensitivity analysis of included studies for respiratory manifestations. (A) Sensitivity analysis of included studies for cough. (B) Sensitivity analysis of included studies for sore throat. (C) Sensitivity analysis of included studies for chest pain. The studies included were as follows: Aguilar *et al* (24), Alvarez-Falconi *et al* (25), Alva-Urcia *et al* (26), Azevedo *et al* (11), Benitez *et al* (34), Cardoso *et al* (16), Carvalho *et al* (17), Castillo *et al* (27), Durango-Chavez *et al* (28), Martins-Luna *et al* (29), Pinheiro *et al* (13), Silva-Caso *et al* (30), Watts *et al* (31) 95% CI, 95% confidence interval.

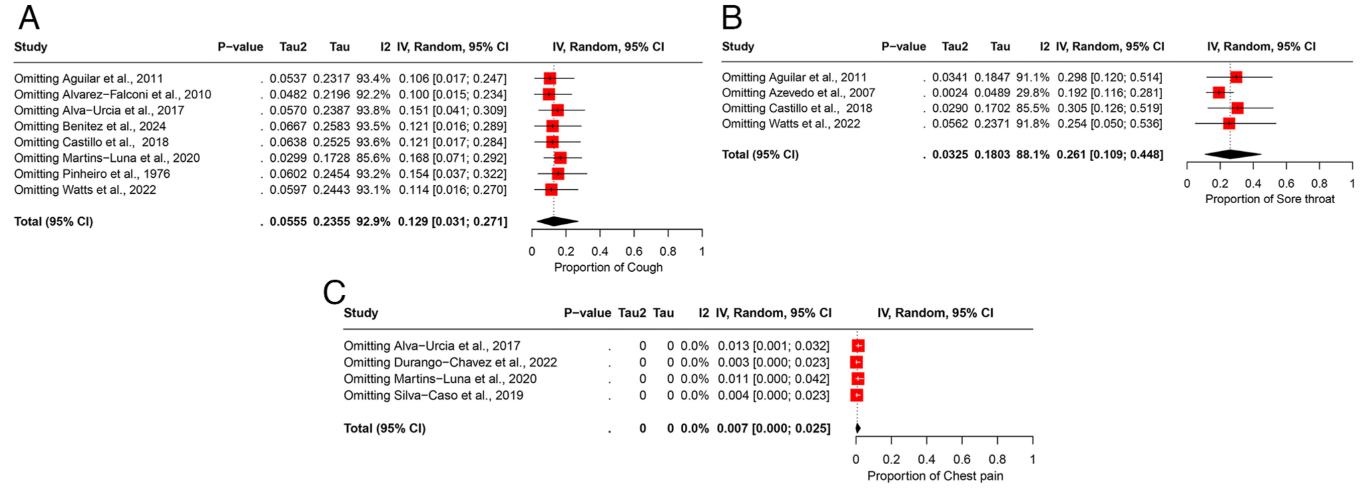

Figure S5. Sensitivity analysis of included studies for gastrointestinal manifestations. (A) Sensitivity analysis of Included studies for nausea or vomiting. (B) Sensitivity analysis of included studies for abdominal pain. (C) Sensitivity analysis of included studies for diarrhoea. (D) Sensitivity analysis of included studies for loss of appetite. (E) sensitivity analysis of included studies for odynophagia. The studies included were as follows: Aguilar *et al* (24), Alvarez-Falconi *et al* (25), Alva-Urcia *et al* (26), Azevedo *et al* (11), Benitez *et al* (34), Cardoso *et al* (16), Carvalho *et al* (17), Castillo *et al* (27), Ciuoderis *et al* (36), Cola *et al* (18), Cravo *et al* (19), da Costa *et al* (20), de Lima *et al* (21), de Melo Iani *et al* (22), Durango-Chavez *et al* (28), Gaillet *et al* (32), Gourjault *et al* (33), Gravier *et al* (35), Martins-Luna *et al* (29), Moreira *et al* (2), Morrison *et al* (37), Mourão *et al* (23), Naveca *et al* (12), Pinheiro *et al* (13), Silva-Caso *et al* (30), Vasconcelos *et al* (14), Vasconcelos *et al* (15), Watts *et al* (31). 95% CI, 95% confidence interval.

A

Study	P-value	Tau2	Tau	I2	IV, Random, 95% CI
Omitting Aguilar et al., 2011	.	0.1319	0.3631	98.3%	0.278 [0.156; 0.418]
Omitting Alvarez-Falconi et al., 2010	.	0.1333	0.3652	98.3%	0.286 [0.162; 0.428]
Omitting Alva-Urcia et al., 2017	.	0.1316	0.3627	98.3%	0.290 [0.167; 0.431]
Omitting Azevedo et al., 2007	.	0.1197	0.3459	98.1%	0.308 [0.186; 0.444]
Omitting Benitez et al., 2024	.	0.1294	0.3597	98.2%	0.299 [0.174; 0.440]
Omitting Cardoso et al., 2015	.	0.1312	0.3623	98.3%	0.292 [0.169; 0.431]
Omitting Carvalho et al., 2022	.	0.1330	0.3647	98.3%	0.288 [0.164; 0.430]
Omitting Castillo et al., 2018	.	0.1339	0.3660	98.3%	0.275 [0.152; 0.416]
Omitting Ciuoderis et al., 2022	.	0.1373	0.3705	98.3%	0.284 [0.159; 0.428]
Omitting Cola et al., 2025	.	0.1353	0.3678	98.3%	0.279 [0.156; 0.422]
Omitting Cravo et al., 2021	.	0.1367	0.3698	98.3%	0.288 [0.162; 0.432]
Omitting daCosta et al., 2017	.	0.1314	0.3625	98.3%	0.288 [0.165; 0.427]
Omitting de Lima et al., 2025	.	0.1440	0.3795	98.3%	0.278 [0.151; 0.425]
Omitting de Melo Iani et al., 2025	.	0.1136	0.3371	98.0%	0.308 [0.189; 0.441]
Omitting Durango-Chavez et al., 2022	.	0.1225	0.3500	98.1%	0.305 [0.182; 0.443]
Omitting Gaillet et al., 2021	.	0.1322	0.3636	98.3%	0.288 [0.164; 0.429]
Omitting Gourjault et al., 2025	.	0.1313	0.3624	98.3%	0.282 [0.161; 0.421]
Omitting Gravier et al., 2025	.	0.1287	0.3587	98.2%	0.299 [0.174; 0.439]
Omitting Martins-Luna et al., 2020	.	0.1390	0.3728	98.3%	0.281 [0.156; 0.425]
Omitting Moreira et al., 2024	.	0.1317	0.3629	98.3%	0.288 [0.164; 0.428]
Omitting Morrison et al., 2024	.	0.1320	0.3634	98.3%	0.289 [0.165; 0.430]
Omitting Mourāão et al., 2009	.	0.1144	0.3382	98.0%	0.308 [0.188; 0.441]
Omitting Naveca et al., 2024	.	0.0959	0.3097	96.4%	0.273 [0.167; 0.393]
Omitting Pinheiro et al., 1976	.	0.1326	0.3642	98.3%	0.294 [0.169; 0.436]
Omitting Silva-Caso et al., 2019	.	0.1338	0.3657	98.3%	0.282 [0.158; 0.424]
Omitting Vasconcelos et al., 1989	.	0.1330	0.3647	98.3%	0.291 [0.166; 0.433]
Omitting Vasconcelos et al., 2009	.	0.1374	0.3706	98.3%	0.286 [0.160; 0.430]
Omitting Watts et al., 2022	.	0.1351	0.3675	98.3%	0.281 [0.157; 0.423]
**Total (95% CI)**	**.**	**0.1309**	**0.3619**	**98.3%**	**0.289 [0.167; 0.427]**

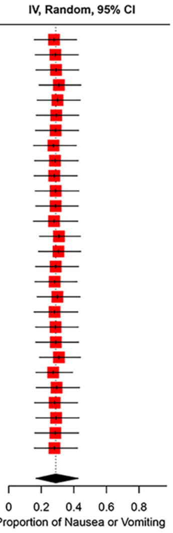

B

Study	P-value	Tau2	Tau	I2	IV, Random, 95% CI
Omitting Aguilar et al., 2011	.	0.0344	0.1854	88.9%	0.151 [0.081; 0.235]
Omitting Alvarez-Falconi et al., 2010	.	0.0353	0.1879	88.9%	0.154 [0.082; 0.241]
Omitting Alva-Urcia et al., 2017	.	0.0360	0.1896	89.4%	0.176 [0.100; 0.265]
Omitting Benitez et al., 2024	.	0.0398	0.1995	89.5%	0.171 [0.092; 0.266]
Omitting Carvalho et al., 2022	.	0.0377	0.1941	89.6%	0.171 [0.094; 0.263]
Omitting Castillo et al., 2018	.	0.0295	0.1717	86.7%	0.145 [0.079; 0.224]
Omitting Ciuoderis et al., 2022	.	0.0329	0.1813	87.4%	0.152 [0.082; 0.236]
Omitting Cravo et al., 2021	.	0.0387	0.1966	89.0%	0.175 [0.096; 0.270]
Omitting daCosta et al., 2017	.	0.0351	0.1874	89.3%	0.156 [0.085; 0.240]
Omitting Durango-Chavez et al., 2022	.	0.0347	0.1864	88.1%	0.179 [0.102; 0.269]
Omitting Gaillet et al., 2021	.	0.0370	0.1923	89.6%	0.163 [0.088; 0.253]
Omitting Gourjault et al., 2025	.	0.0334	0.1829	88.8%	0.150 [0.083; 0.231]
Omitting Martins-Luna et al., 2020	.	0.0356	0.1886	88.0%	0.178 [0.100; 0.269]
Omitting Morrison et al., 2024	.	0.0360	0.1897	89.3%	0.157 [0.084; 0.245]
Omitting Silva-Caso et al., 2019	.	0.0381	0.1951	89.6%	0.170 [0.093; 0.263]
Omitting Vasconcelos et al., 1989	.	0.0369	0.1922	89.2%	0.159 [0.085; 0.248]
Omitting Vasconcelos et al., 2009	.	0.0394	0.1984	89.2%	0.174 [0.095; 0.269]
**Total (95% CI)**	**.**	**0.0359**	**0.1894**	**88.9%**	**0.163 [0.091; 0.249]**

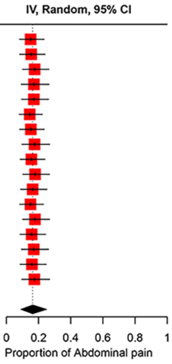

C

Study	P-value	Tau2	Tau	I2	IV, Random, 95% CI
Omitting Aguilar et al., 2011	.	0.0272	0.1648	90.9%	0.171 [0.098; 0.256]
Omitting Alvarez-Falconi et al., 2010	.	0.0305	0.1747	91.6%	0.193 [0.112; 0.287]
Omitting Alva-Urcia et al., 2017	.	0.0293	0.1712	91.6%	0.190 [0.113; 0.281]
Omitting Azevedo et al., 2007	.	0.0135	0.1163	81.8%	0.151 [0.096; 0.214]
Omitting Benitez et al., 2024	.	0.0326	0.1804	91.6%	0.197 [0.113; 0.294]
Omitting Carvalho et al., 2022	.	0.0303	0.1740	91.6%	0.189 [0.110; 0.283]
Omitting Ciuoderis et al., 2022	.	0.0325	0.1804	91.4%	0.189 [0.106; 0.285]
Omitting Cravo et al., 2021	.	0.0331	0.1820	91.5%	0.201 [0.116; 0.300]
Omitting daCosta et al., 2017	.	0.0290	0.1702	91.6%	0.186 [0.111; 0.274]
Omitting Gaillet et al., 2021	.	0.0287	0.1694	91.3%	0.179 [0.103; 0.268]
Omitting Gourjault et al., 2025	.	0.0284	0.1685	91.4%	0.180 [0.107; 0.267]
Omitting Morrison et al., 2024	.	0.0261	0.1614	90.5%	0.167 [0.096; 0.251]
Omitting Naveca et al., 2024	.	0.0320	0.1789	86.1%	0.199 [0.115; 0.296]
Omitting Pinheiro et al., 1976	.	0.0314	0.1771	91.5%	0.190 [0.109; 0.285]
Omitting Watts et al., 2022	.	0.0284	0.1684	90.7%	0.178 [0.102; 0.267]
**Total (95% CI)**	**.**	**0.0287**	**0.1693**	**91.0%**	**0.184 [0.110; 0.271]**

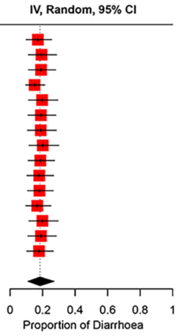

D

Study	P-value	Tau2	Tau	I2	IV, Random, 95% CI
Omitting Aguilar et al., 2011	.	0.0920	0.3034	95.8%	0.383 [0.211; 0.571]
Omitting Alvarez-Falconi et al., 2010	.	0.1122	0.3350	96.4%	0.443 [0.245; 0.650]
Omitting Alva-Urcia et al., 2017	.	0.1067	0.3267	96.4%	0.452 [0.259; 0.652]
Omitting Benitez et al., 2024	.	0.1101	0.3318	96.1%	0.471 [0.272; 0.676]
Omitting Carvalho et al., 2022	.	0.1117	0.3343	96.4%	0.444 [0.247; 0.651]
Omitting Castillo et al., 2018	.	0.1084	0.3292	96.2%	0.423 [0.230; 0.627]
Omitting Cravo et al., 2021	.	0.0597	0.2444	92.8%	0.496 [0.342; 0.650]
Omitting Durango-Chavez et al., 2022	.	0.1112	0.3335	96.1%	0.425 [0.230; 0.632]
Omitting Gaillet et al., 2021	.	0.1087	0.3298	96.4%	0.452 [0.256; 0.655]
Omitting Martins-Luna et al., 2020	.	0.1049	0.3239	95.7%	0.420 [0.231; 0.622]
Omitting Silva-Caso et al., 2019	.	0.1124	0.3352	96.4%	0.438 [0.241; 0.646]
Omitting Vasconcelos et al., 1989	.	0.1059	0.3254	96.2%	0.475 [0.279; 0.675]
**Total (95% CI)**	**.**	**0.1042**	**0.3227**	**96.1%**	**0.443 [0.260; 0.635]**

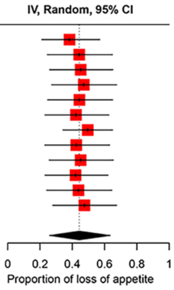

E

Study	P-value	Tau2	Tau	I2	IV, Random, 95% CI
Omitting Alvarez-Falconi et al., 2010	.	0.0123	0.1111	78.2%	0.304 [0.202; 0.417]
Omitting Alva-Urcia et al., 2017	.	0.0263	0.1622	89.5%	0.277 [0.151; 0.423]
Omitting Ciuoderis et al., 2022	.	0.0462	0.2151	91.6%	0.213 [0.069; 0.403]
Omitting Durango-Chavez et al., 2022	.	0.0347	0.1863	89.4%	0.185 [0.064; 0.346]
Omitting Martins-Luna et al., 2020	.	0.0457	0.2139	91.1%	0.195 [0.058; 0.381]
Omitting Silva-Caso et al., 2019	.	0.0381	0.1952	91.6%	0.204 [0.073; 0.374]
**Total (95% CI)**	**.**	**0.0319**	**0.1785**	**89.6%**	**0.229 [0.109; 0.374]**

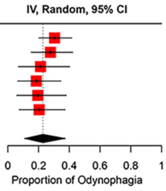

Figure S6. Sensitivity analysis of included studies for musculoskeletal manifestations. (A) Sensitivity analysis of included studies for myalgia. (B) Sensitivity analysis of included studies for arthralgia. (C) Sensitivity analysis of included studies for back pain. The studies included were as follows: Aguilar *et al* (24), Alvarez-Falconi *et al* (25), Alva-Urcia *et al* (26), Azevedo *et al* (11), Benitez *et al* (34), Cardoso *et al* (16), Carvalho *et al* (17), Castillo *et al* (27), Ciuoderis *et al* (36), Cola *et al* (18), Cravo *et al* (19), da Costa *et al* (20), de Lima *et al* (21), de Melo Iani *et al* (22), Durango-Chavez *et al* (28), Gaillet *et al* (32), Gourjault *et al* (33), Gravier *et al* (35), Martins-Luna *et al* (29), Moreira *et al* (2), Morrison *et al* (37), Mourão *et al* (23), Naveca *et al* (12), Pinheiro *et al* (13), Silva-Caso *et al* (30), Vasconcelos *et al* (14), Vasconcelos *et al* (15), Watts *et al* (31) 95% CI, 95% confidence interval.

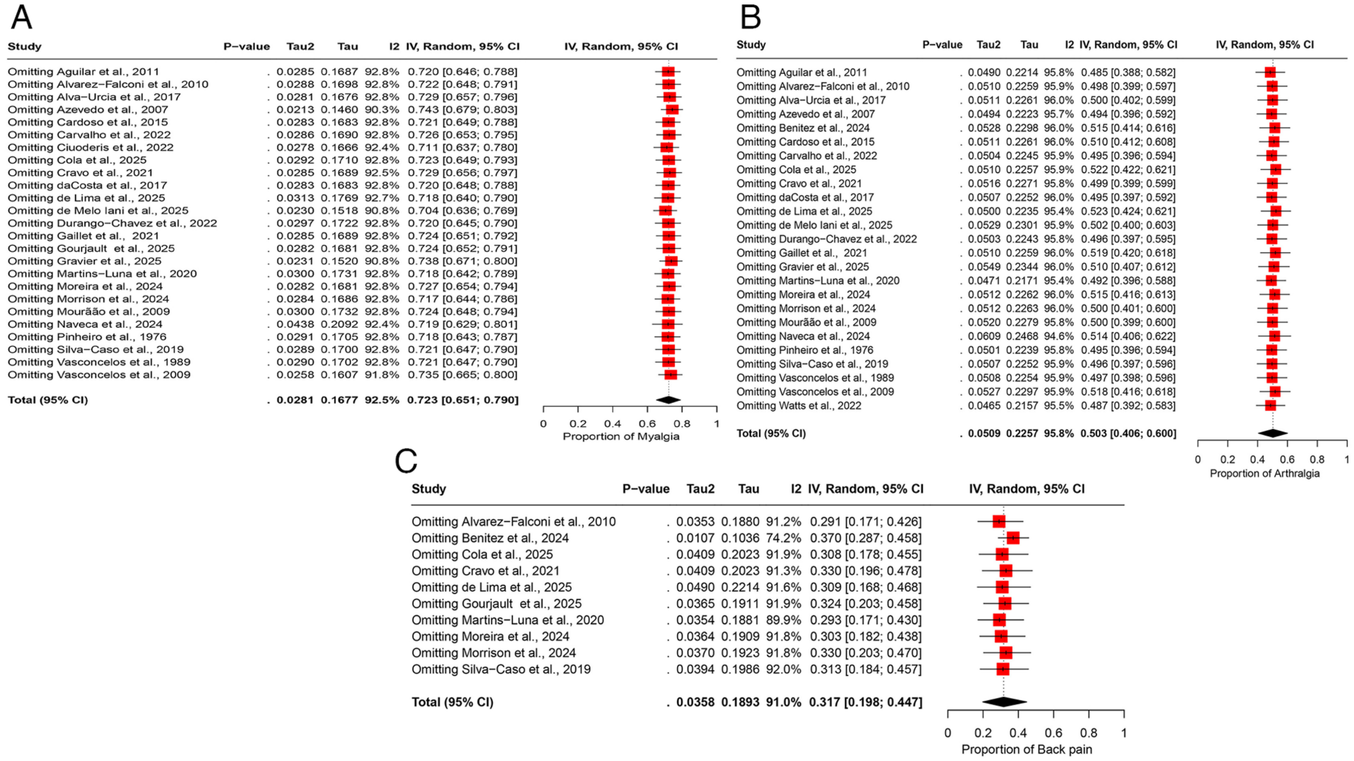

Figure S7. Sensitivity analysis of included studies for dermatological manifestations. (A) Sensitivity analysis of included studies for skin rash. (B) Sensitivity analysis of included studies for petechiae. The studies included were as follows: Alvarez-Falconi *et al* (25), Alva-Urcia *et al* (26), Cardoso *et al* (16), Carvalho *et al* (17), Castillo *et al* (27), Ciuoderis *et al* (36), Cola *et al* (18), Cravo *et al* (19), da Costa *et al* (20), de Lima *et al* (21), Durango-Chavez *et al* (28), Gaillet *et al* (32), Gourjault *et al* (33), Martins-Luna *et al* (29), Moreira *et al* (2), Morrison *et al* (37), Mourão *et al* (23), Naveca *et al* (12), Silva-Caso *et al* (30), Vasconcelos *et al* (14), Watts *et al* (31) 95% CI, 95% confidence interval.

A

Study	P−value	Tau2	Tau	I2	IV, Random, 95% CI
Omitting Alvarez−Falconi et al., 2010	.	0.0500	0.2235	95.9%	0.197 [0.107; 0.303]
Omitting Alva−Urcia et al., 2017	.	0.0488	0.2208	95.9%	0.196 [0.108; 0.300]
Omitting Carvalho et al., 2022	.	0.0477	0.2185	95.7%	0.176 [0.092; 0.277]
Omitting Castillo et al., 2018	.	0.0503	0.2243	95.9%	0.186 [0.098; 0.292]
Omitting Ciuoderis et al., 2022	.	0.0520	0.2280	95.9%	0.187 [0.098; 0.295]
Omitting Cravo et al., 2021	.	0.0432	0.2077	95.0%	0.170 [0.090; 0.266]
Omitting daCosta et al., 2017	.	0.0476	0.2182	95.8%	0.173 [0.092; 0.272]
Omitting de Lima et al., 2025	.	0.0586	0.2421	95.9%	0.196 [0.100; 0.312]
Omitting Durango−Chavez et al., 2022	.	0.0481	0.2193	95.6%	0.177 [0.092; 0.280]
Omitting Gaillet et al., 2021	.	0.0490	0.2215	95.9%	0.185 [0.099; 0.289]
Omitting Gourjault et al., 2025	.	0.0449	0.2118	95.6%	0.158 [0.082; 0.251]
Omitting Morrison et al., 2024	.	0.0480	0.2190	95.8%	0.176 [0.092; 0.278]
Omitting Mourãão et al., 2009	.	0.0370	0.1925	94.2%	0.163 [0.089; 0.252]
Omitting Naveca et al., 2024	.	0.0376	0.1939	90.9%	0.195 [0.115; 0.290]
Omitting Silva−Caso et al., 2019	.	0.0468	0.2163	95.6%	0.173 [0.090; 0.273]
Omitting Vasconcelos et al., 2009	.	0.0529	0.2301	95.9%	0.197 [0.105; 0.308]
Omitting Watts et al., 2022	.	0.0503	0.2242	95.9%	0.185 [0.097; 0.291]
**Total (95% CI)**	**.**	**0.0482**	**0.2196**	**95.7%**	**0.182 [0.099; 0.281]**

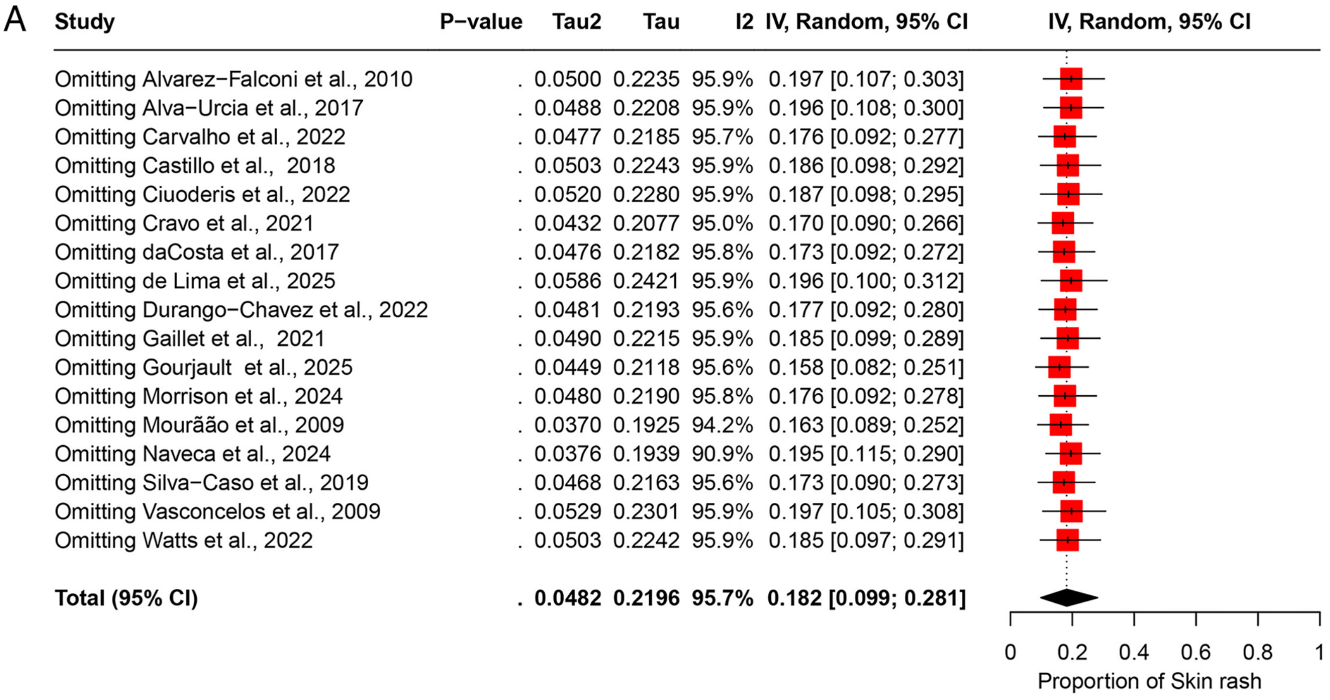

B

Study	P−value	Tau2	Tau	I2	IV, Random, 95% CI
Omitting Alvarez−Falconi et al., 2010	.	0.0131	0.1143	82.4%	0.023 [0.001; 0.062]
Omitting Cardoso et al., 2015	.	0.0118	0.1085	81.6%	0.026 [0.005; 0.060]
Omitting Ciuoderis et al., 2022	.	0.0108	0.1037	78.0%	0.029 [0.005; 0.067]
Omitting Cola et al., 2025	.	0.0129	0.1137	81.6%	0.019 [0.000; 0.056]
Omitting Cravo et al., 2021	.	0.0126	0.1124	80.5%	0.018 [0.000; 0.054]
Omitting de Lima et al., 2025	.	0.0158	0.1257	82.2%	0.024 [0.001; 0.067]
Omitting Durango−Chavez et al., 2022	.	0.0133	0.1152	81.6%	0.026 [0.002; 0.066]
Omitting Martins−Luna et al., 2020	.	0.0129	0.1137	80.5%	0.027 [0.002; 0.067]
Omitting Moreira et al., 2024	.	0.0125	0.1119	82.3%	0.026 [0.003; 0.063]
Omitting Mourãão et al., 2009	.	0.0051	0.0714	62.1%	0.012 [0.000; 0.035]
Omitting Silva−Caso et al., 2019	.	0.0122	0.1106	81.3%	0.027 [0.003; 0.066]
**Total (95% CI)**	**.**	**0.0121**	**0.1098**	**80.4%**	**0.023 [0.002; 0.058]**

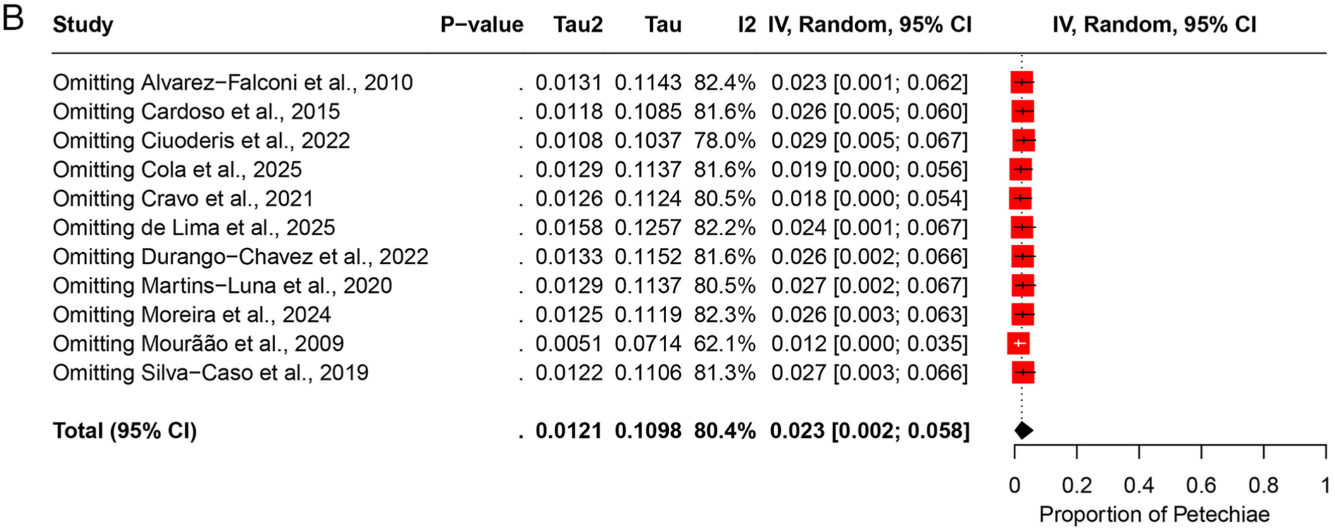

Figure S8. Funnel plot assessing publication bias for fever among the included studies

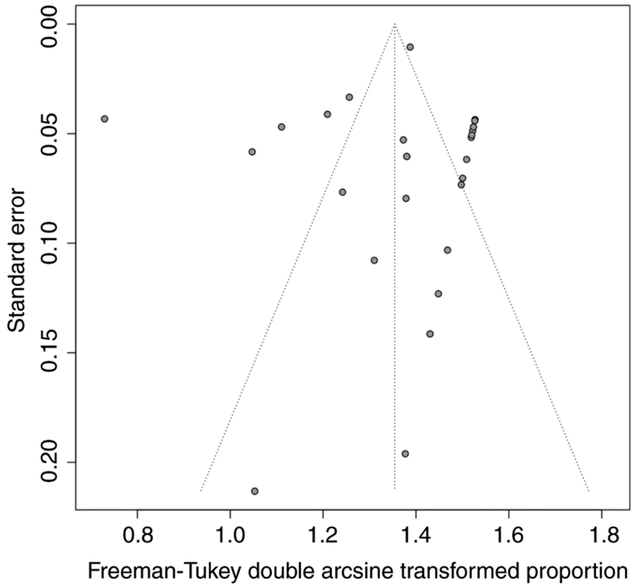

Supplement: Sensitivity analysis of included studies for general or systemic manifestations. (A) Sensitivity analysis of included studies for fever. (B) Sensitivity Analysis of Included studies for malaise or fatigue. (C) Sensitivity analysis of Included studies for chills. (D) Sensitivity analysis of included  [file Supplementary_Data1.pdf]
