# Supplementary material for: Clinical manifestations of Oropouche virus infection: A systematic review and meta-analysis
Source: Med Int (Lond). 2025 Aug 27;5(6):67. doi: 10.3892/mi.2025.266 (PMC12416135; doi:10.3892/mi.2025.266)
Supplement: The detailed search strategy for each database. [file Supplementary_Data2.pdf]

Table SI. The detailed search strategy for each database.

Database	Search query	Search results
PubMed	(‘Oropouche orthobunyavirus’[Title/Abstract] OR ‘Oropouche fever’[Title/Abstract] OR ‘Oropouche virus’[Title/Abstract] OR oropouche[Title/Abstract] OR OROV[Title/Abstract] OR ‘Oropouche virus infection’[Title/Abstract] OR ‘Oropouche fever virus’[Title/Abstract] OR ‘Oropouche virus disease’[Title/Abstract] OR ‘Orthobunyavirus Oropouche’[Title/Abstract] OR "Oropouche Orthobunyavirus infection’[Title/Abstract] OR ‘Oropouche Virus (OROV)’[Title/Abstract] OR ‘Oropouche virus (OROV)’[Title/Abstract])	361
Embase	‘oropouche orthobunyavirus’/exp OR ‘oropouche orthobunyavirus’ OR ‘oropouche fever’/exp OR ‘oropouche fever’ OR ‘oropouche virus’/exp OR ‘oropouche virus’ OR oropouche OR ‘oropouche virus infection’/exp OR ‘oropouche virus infection’ OR ‘oropouche fever virus’ OR ‘oropouche virus disease’ OR ‘orthobunyavirus oropouche’ OR ‘oropouche orthobunyavirus infection’ OR ‘oropouche virus (orov)’ OR ‘orov’	447
Web of Science	TS=(‘Oropouche orthobunyavirus’ OR ‘Oropouche fever’ OR ‘Oropouche virus’ OR oropouche OR OROV OR ‘Oropouche virus infection’ OR ‘Oropouche fever virus’ OR ‘Oropouche virus disease’ OR ‘Orthobunyavirus Oropouche’ OR ‘Oropouche Orthobunyavirus infection’ OR ‘Oropouche Virus (OROV)’ OR ‘Oropouche virus (OROV)’ OR ‘OROV’)	363

Table SII. Quality appraisal of studies using the modified Newcastle-Ottawa Scale.

First author, year of publication	Representativeness	Sample size 500	Definition of test used	Ascertainment of event	Total	(Refs.)[a]
Aguilar, 2011	2	0	2	1	5	(24)
Alvarez-Falconi, 2010	2	0	2	1	5	(25)
Alva-Urcia, 2017	2	0	2	1	5	(26)
Azevedo, 2007	2	0	2	1	5	(11)
Benitez, 2024	2	0	2	1	5	(34)
Cardoso, 2015	2	0	2	1	5	(16)
Carvalho, 2022	2	0	2	1	5	(17)
Castillo, 2018	2	0	2	1	5	(27)
Ciuoderis, 2022	2	0	2	1	5	(36)
Cravo, 2021	2	0	2	1	5	(19)
da Costa, 2017	2	0	2	1	5	(20)
de Lima, 2025	2	0	2	1	5	(21)
de Melo Iani, 2025	2	0	2	1	5	(22)
Durango-Chavez, 2022	2	0	2	1	5	(28)
Gaillet, 2021	2	0	2	1	5	(32)
Gravier, 2025	2	0	2	1	5	(35)
Martins-Luna, 2020	2	0	2	1	5	(29)
Moreira, 2024	2	0	2	1	5	(2)
Morrison, 2024	2	0	2	1	5	(37)
Mourãão, 2009	2	0	2	1	5	(23)
Naveca, 2024	2	1	2	1	6	(12)
Pinheiro, 1976	1	0	2	1	4	(13)
Silva-Caso, 2019	2	0	2	1	5	(30)
Vasconcelos, 1989	1	0	2	1	4	(14)
Vasconcelos, 2009	1	0	2	1	4	(15)
Watts, 2022	2	0	2	1	5	(31)

[a]Reference citations can be found in the reference list in the main manuscript.

Table SIII. Methodological quality assessment of case series/reports using the tool described in the study by Murad *et al* (10)[a].

First author, year of publication	Q1: Selection	Q2: Exposure ascertained	Q3: Outcome ascertained	Q4: Follow-up for outcomes	Q5: Sufficient reporting	(Refs.)[a]
Cola, 2025	Yes	Yes	Yes	Yes	Yes	(18)
Gourjault, 2025	Yes	Yes	Yes	Yes	Yes	(33)

[a]Reference citations can be found in the reference list in the main manuscript.
